# Confirmation by Robustness Analysis: A Bayesian Account

**DOI:** 10.1007/s10670-022-00537-7

**Published:** 2022-05-11

**Authors:** Lorenzo Casini, Jürgen Landes

**Affiliations:** 1 Institute of Economics, Sant’Anna School of Advanced Studies, Pisa, Italy; 2 Munich Center for Mathematical Philosophy, Open Science Center, LMU Munich, Munich, Germany

**Keywords:** Robustness analysis, Minimal models, Agent-based models, Confirmation, Variety of evidence, Stylized facts of finance

## Abstract

Some authors claim that minimal models have limited epistemic value (Fumagalli, 2016; Grüne-Yanoff, 2009a). Others defend the epistemic benefits of modelling by invoking the role of robustness analysis for hypothesis confirmation (see, e.g., Levins, 1966; Kuorikoski et al., 2010) but such arguments find much resistance (see, e.g., Odenbaugh & Alexandrova, 2011). In this paper, we offer a Bayesian rationalization and defence of the view that robustness analysis can play a confirmatory role, and thereby shed light on the potential of minimal models for hypothesis confirmation. We illustrate our argument by reference to a case study from macroeconomics. At the same time, we also show that there are cases in which robustness analysis is detrimental to confirmation. We characterize these cases and link them to recent investigations on evidential variety (Landes, 2020b, 2021; Osimani and Landes, forthcoming). We conclude that robustness analysis over minimal models *can* confirm, but its confirmatory value depends on concrete circumstances.

## Introduction

In recent years, philosophy of science has paid much attention to so-called “minimal” models. Minimal models may be informally characterized as models lacking clear world-linking properties: they are highly idealized, not constructed starting from well-confirmed laws or structural identities, not even in the sense of isolating some real causal factor, and not purporting to represent any specific target (see Grüne-Yanoff, 2009a, p. 83). What, if anything, can be learned from such models?

A paradigmatic example of a minimal model is Thomas Schelling’s (1971) checkerboard model. This model is designed to study the unintended emergence of housing segregation patterns out of uncoordinated individual actions. The phenomenon is studied on an artificial grid. Each cell in the grid corresponds to a space which an artificial agent can occupy. Agents, belonging to two groups, are represented by pennies and dimes. They aim to satisfy just one preference, namely, they want to live in a cell whose neighbourhood comprises at least a certain proportion of their own group. At each time step, they can either stay where they are, if their preference is satisfied, or move to a free cell whose neighbourhood satisfies their preference. As a result of simulating the agent’s moves, segregation obtains across many initial distributions of agents on the grid and preference strengths. What can one learn from this model, given that it is built without a specific target in mind, that it misrepresents human interactions and decision making in many ways, and that it idealizes any causal factor possibly responsible for segregation?

Philosophers’ opinions tend to be sceptical. The received view on the epistemic benefits of highly idealized models is that these models are useful to explore a theory’s consequences but—unlike experiments (Grüne-Yanoff, 2009b; Guala, 2002; Morgan, 2005)—they cannot establish any hypothesis about the real world (see, e.g., Hausman, 1992, ch. 5). With few exceptions (e.g., Casini, 2014; Knuuttila, 2009; Sugden, 2000), similar claims have been made with regard to *minimal* models. Minimal models can establish possibilities and impossibilities (Grüne-Yanoff, 2009a), that is, what *may* or may *not* be the case; however, they cannot establish what *is* the case. Some (Fumagalli, 2016) go as far as claiming that minimal models do not grant any learning at all, precisely due to their lack of clear world-linking properties. In other words, the received view is that minimal models cannot serve to confirm any empirical hypothesis.^1^

A reply to which an advocate of the confirmatory use of minimal models could resort is to say that *one* minimal model can*not* confirm, but *several* of them *can*. The idea is that results obtained from a single minimal model may depend on any of the idealized and possibly false assumptions of the model and/or on the tractability constraints introduced when building the model. Thus, one cannot exclude that it is those assumptions and constraints, rather than any hypothesis the modeller would like to confirm, which explain the result. However, the same is not true of a collection of such models, which all make the same to-be-tested hypothesis, but otherwise vary all other assumptions and constraints, in order to rule out that they are necessary to generate the results, and thus to lend support to the target hypothesis. This strategy is often referred to in the literature as “robustness analysis” (RA).^2^ The rationale of RA is the following: suppose a number of models share one “core” assumption, but differ with respect to their auxiliary assumptions (none of which being more realistic or justified than any other); then, if all models determine the same result, the result does not hinge on those auxiliary assumptions, but is explained by (among other facts) the core assumption. In Richard Levins’ words,

if these models, despite their different assumptions, lead to similar results, we have what we can call a robust theorem that is relatively free of the details of the model. Hence, our truth is the intersection of independent lies. (Levins, 1966, p. 20)

Since Levins, who first proposed this idea, others have attempted to defend RA’s confirmatory value, among whom Wimsatt (1981, 1987), Weisberg (2006), Kuorikoski et al. (2010, 2012), Lloyd (2015), and most recently, Fuller and Schulz (2021), Sakai (2020), and Boge (forthcoming) as well as Schupbach (2022, Sect. 3.3).

Yet, the view that model exploration is conducive to confirmation has encountered much resistance (see, e.g., Orzack & Sober 1993; Sugden 2000; Odenbaugh & Alexandrova 2011; Fumagalli 2016 Lisciandra 2016; Stegenga & Menon 2017). Exploring the behaviour of a model by varying its assumptions may be useful to make more transparent its consequences; yet, so the objection goes, the mere fact that certain conclusions are robustly derivable from certain assumptions across variation in other assumptions is insufficient to confirm that the former assumptions are true of the real world. Following Odenbaugh and Alexandrova (2011), the worries about the confirmatory value of RA may be perspicuously grouped into two categories. First, there are worries about *undischarged idealizations*:

By and large only some, not all, of the idealizations of models are discharged by robustness analysis. As a result many robust theorems praised by theoreticians remain empirically questionable and thus explanatorily weak. (Odenbaugh & Alexandrova, 2011, p. 759)

And second, there are worries about the *non-independence of the assumptions*:

Robustness analysis crucially depends on showing that the assumptions of different models are independent of one another. However, [...] reports of their independence have been greatly exaggerated. (*ibid.*)

In this paper, we produce a rationalization of the view that minimal models *can* confirm, by defending the view that RA on minimal models *can* increase the confirmation that each model individually lends to some target hypothesis. This rationalization will allow us to address the above worries. Our argument is embedded in a Bayesian framework, where confirmation depends on an increase in the probability of a hypothesis in the light of the evidence.

Our case is buttressed by a case study from macroeconomics. The case in question is the recent explanation of certain stable statistical features of the time series of financial prices, which go under the name of “stylized facts of finance”. The explanation is advanced by non-orthodox economists, who reject a standard macroeconomic assumption, the so-called rational expectation hypothesis, which maintains that agents are alike—or homogeneous—in their having rational expectations. More precisely, we look at two models of the asset pricing mechanism that, albeit minimal, adequately recover a number of stylized facts, viz. a model that views the market as analogous to a fluid undergoing phase transition (Lux & Marchesi, 1999, 2000), and a model that views the market as a population subject to natural selection (Arthur et al., 1997, LeBaron et al., 1999). We argue that the evidence from both models can confirm the hypothesis that the agents’ heterogeneity is necessary to the stylized facts.

At the same time, we also report cases, in which a variation of modelling assumptions is detrimental to confirmation. The availability of multiple sources of evidence, or “evidential variety”, was long thought to be a universal boon for confirmation. This, however, is no longer the case (Bovens & Hartmann, 2003). Drawing on literature on the so-called Variety of Evidence Thesis (Landes, 2020b, 2021; Osimani and Landes, forthcoming), we show that there are a number of cases in which replication is more confirmatory than variation, if auxiliary assumptions of different models are not independent from one another. This means that, although RA can confirm, it is not always the most-confirming strategy. Finally, in addition to the variation of auxiliaries, which is the object of Odenbaugh and Alexandrova’s objections, we also consider the variation (or different implementation) of the hypothesis to be confirmed while the auxiliaries are held fixed, which too falls under the umbrella of RA as variation of modelling assumptions. In sum, we investigate the conditions for variation to confirm more than replication—in short, for RA to be confirmatory—in both cases.

Two general points should be made from the outset about our argument. First, we are concerned with providing possibility results. We show that in certain scenarios there is confirmation (or disconfirmation) by establishing certain inequalities. In order to establish these inequalities, we motivate and impose constraints on a Bayesian prior probability function inspired by our case study. Our conclusions are general in that they apply to a class of prior probability functions and in that the model provided may be easily reinterpreted with reference to other phenomena. We are not interested in *amounts* of confirmation (or disconfirmation). Whilst wide ranges of parameter values suffice for confirmation, specific amounts of confirmation depend on specific values. A justification of such values would require a lengthy discussion. Moreover, it would depend on the details of the case study at hand, and thus not necessarily translate to other cases. Given the general nature of our analysis, thus, a discussion of the strength of confirmation is not only difficult but also beyond the scope of this paper.

Second, we are interested in rationalizing the practice of RA in those scientific communities, whose efforts are directed at developing and exploring models of target systems, since the direct inspection of such targets is either difficult (e.g., economics, paleontology, archaeology^3^) or impossible (e.g., cosmology, theories of quantum gravity^4^). This is not to say that these communities are just concerned with hypothesis confirmation. In particular, scientists may use RA for many reasons, from theoretical explorations, to personal preferences, to a publish or perish culture incentivising publication, etc. We are not concerned with revealing what fraction of scientists is driven by one purpose rather than another. Whilst believing that hypothesis confirmation is an important driver, we also believe that an investigation of the scientists’ motivations for RA would require an entirely different kind of study, based on interviews, extensive literature reviews, etc. Here, we rather demonstrate that, if the scientists’ goal were indeed confirmation, the use of RA to this end could be rationally justified.

The rest of the paper is organized as follows. In Sect. 2, we introduce our case study. In Sect. 3, we present our model for a Bayesian reconstruction of confirmation by RA. Section 4 contains the formal analysis of the model and the explanations of our results. Section 5 concludes the paper.

## What Explains the Stylized Facts of Finance?

### The Discontent with Rational Expectations

Our case study is best introduced against the backdrop of the dominant paradigm in macroeconomics, namely the “neoclassical” theory and more recent developments of it. The theory is based on the fundamental assumption of rational expectations, or rational expectation hypothesis (REH), which holds that agents choose among alternative courses of action the one that maximizes their expected utility or profits. This entails that, in the financial market, given full information about the assets (e.g., bonds, stocks, other financial instruments), agents sell those assets, which are priced above their fundamental values, and buy those assets, which are priced below their fundamental values. Since agents are equal (or “homogeneous”) in this respect, their aggregate behavior is reducible to the behavior of one representative agent. Given that prices immediately and unbiasedly reflect the behaviour of rational agents, the market is at equilibrium (demand and offer balance each other, such that the market clears), and the equilibrium is optimal (market prices are always the immediate and unbiased reflection of the underlying fundamental values of the assets)—which is known as the efficient market hypothesis (EMH). Variations in price are thus uniquely imputable to exogenous shocks (typically, technological innovations), because all information on how the shocks affect the fundamentals is learned by the agents, who immediately exploit any profit opportunity due to temporary differences between assets’ prices and their fundamentals, such that prices quickly revert to the underlying fundamentals. Given EMH and the unpredictable character of the shocks, prices should follow a random walk.

A weakness of the neoclassical theory, as applied to financial markets, is that it is unable to explain bubbles and crashes and more general statistical features of the time series of prices. Such features are known as “stylized facts”, due to their qualitative nature and their stability across different instruments, times and markets.^5^ The neoclassical theory correctly predicts one such fact, namely that the unconditional distribution of returns (i.e., relative price changes) at low (e.g., annual) frequencies is roughly Gaussian, which entails that the direction of returns is generally unpredictable, in agreement with the random walk hypothesis. However, it cannot explain other stylized facts, for instance why the unconditional distribution of returns at higher (e.g., daily) frequencies is *fat-tailed* (i.e., with too many observations near the mean and in the tails, and too few in the mid range). Moreover, the theory does not explain certain features of the conditional distribution of returns: returns at different times are not independent (*volatility persistence*), and price changes of the same size tend to follow one another (*volatility clustering*). In particular, the decay of the autocorrelation of returns is slower than predicted by the theory, meaning that information about price trends is useful. This contradicts EMH, which entails that information on past prices should be discounted given information on current prices, because current prices correctly reflect the underlying fundamentals, and are thus the best predictors of future prices. Other stylized facts have been reported, but the above list will suffice for present purposes.

Ideally, one would like to explain all of these stylized facts. Moreover, policy makers are particularly interested in controlling financial crises. To achieve these (and other) goals, it has become popular to supplement the neoclassical tenets that output changes are caused by shocks in technology and that markets are efficient (at least in the long run) with the Keynesian idea that short-run fluctuations—and, consequently, inefficient equilibria—are still possible, due to market imperfections. The combination of these views is known as “new neoclassical synthesis” (see, e.g., Woodford, 2009). The models informed by this refined theory—viz. dynamic stochastic general equilibrium (DSGE) models—purport to predict the dynamics of aggregate phenomena, such as economic growth, business cycles, and the effects of monetary and fiscal policies, starting from microeconomic principles, most notably REH. Central banks have until now used DSGE models to respond to financial crises, whether because of trust or, more simply, for lack of a better alternative.

Different DSGE models underpin correspondingly different accounts of the stylized facts. One example are models allowing for the possibility of “sunspot” equilibria (Cass & Shell, 1983). A sunspot is an exogenous cause of shocks that does not affect the fundamentals but can still affect economic outcomes, when agents have *imperfect information* on what affects the fundamentals and what doesn’t, and coordinate their choices on the sunspot, thereby generating inefficient equilibria with excess volatility. Another example are models introducing “frictions” in the form of “nominal” rigidities, such as “sticky” prices and wages, or financial rigidities, such as bankruptcy costs, and credit and equity rationing. One such model is the financial accelerator model (Bernanke et al., 1996). Firms (borrowers) and banks (lenders) have *asymmetric information* about the real value of the assets. This generates a self-reinforcing process that amplifies the shocks’ effects on the assets’ prices: a shock decreases a firm’s activity, which lowers the asset’s price, which reduces the net worth of the firm’s collateral, which limits the firm’s access to credit, which slows activity, and so on.

A large variety of DSGE models exist (for two reviews, see Milani, 2012 and Dilaver et al., 2018). What is important for our purposes is that: first, although these models may relax this or that neoclassical assumption, they all share the core neoclassical principle, viz. REH; second, they purport to explain the stylized facts of finance in terms of exogenous shocks to the economy; but, third, they have a hard time in accounting for these facts, let alone correctly predict the effect of policies during or after financial crises (see Haldane & Turrell 2018, p. 227). The resistence of many economists to abandon REH in the face of apparent disconfirmations (for a recent example, see Balfoussia et al. 2018, Sect. 7) has been criticized by several influential economists (Kirman, 2010; Krugman, 2011; Romer, 2016; Stiglitz, 2016), who have condemned the effort devoted to save REH by playing with peripheral assumptions, often without much theoretical justification. At the same time, a new research programme is emerging, which moves away from the neoclassical paradigm.

### The Heterogeneity Hypothesis

The new research programme in question is agent-based computational economics (see Tesfatsion, 2002, 2006a, b; on the application to financial macroeconomics, see LeBaron, 2006a, b and Fagiolo & Roventini, 2017). Agent-based computational economics rejects REH. Agents are *heterogeneous*. Since asset prices cannot be calculated by maximizing the utility of a representative agent, population-based models must be replaced by agent-based models, which directly compute the aggregate demand of heterogeneous agents. We now briefly describe two models in this new research programme, which will later serve to illustrate our proposal.

The two models in question aim to reproduce a number of stylized facts by providing idealized—minimal—analogues of the asset pricing mechanism in the financial market. They take inspiration from mechanisms in other domains—viz. phase transition in physics (Lux & Marchesi, 1999, 2000) and natural selection in biology (Arthur et al., 1997, LeBaron et al., 1999)—that are known to generate stylized facts (of a non-financial kind) as a result of self-reinforcing feedbacks—respectively, state transitions catalyzing themselves and genetic traits becoming more and more entrenched—ultimately due to the dishomogeneity among the components of the system—respectively, among the particles’ states and among the individuals’ genetic codes. It is typically assumed that market bubbles and crashes obtain when some force drives the market away from equilibrium, by making price fluctuations become self-reinforcing in the upward or downward direction. The modellers’ conjecture is that, in spite of the obvious diversity between physical and biological systems on the one hand, and financial markets on the other hand, the components’ *heterogeneity* is in both cases essential to the process that generates stylized facts. Consequently, guided by the above analogies (cf. Lux & Marchesi, 1999, p. 498; Arthur et al., 1997, Sect. 5), they represent agents as particles in a fluid undergoing phase transition, where the different states correspond to different groups of agents, in one case, and as populations of genetic codes subject to natural selection, each code representing a trading strategy, in the other case. In both cases, agents are *heterogeneous* and *boundedly rational*. Heterogeneity is realized by different dispositions in the phase transition model, and by different expectations in the evolutionary model. Dispositions and expectations change over time. In the phase transition model, agents *switch* between groups. In the evolutionary model, agents *learn* new strategies. Let us examine the two models in some more detail.

In the phase transition model, traders are divided into two main groups: fundamentalists, who sell (respectively, buy) when the price is above (below) the fundamental value; and chartists (or “noise traders”), subdivided in turn into optimists and pessimists, who buy or sell depending on trends and opinions. Traders can switch between different groups, like particles switch between different states. The number of individuals in each group determines the aggregate excess demand, which results—via the operation of a market maker, or Walrasian auctioneer—in changes in actual price, which in turn affect the agents’ trading attitudes. Changes in fundamental value are governed by a random process. Switching between groups is governed by time-varying probability functions: the fundamentalist-chartist switch depends on a comparison of the respective profits (realized profits for the chartists, expected profits for the fundamentalists); the optimist-pessimist switch depends on an opinion index (representing the average opinion among chartists) and the price trend.

In the evolutionary model, in contrast, each agent is a “theory” of trading rules. Each theory is like a genotype, made of 100 rules. Each rule is like a chromosome, and consists in a set of predictors, comprising a condition part (which may represent fundamental or chartist information) and a forecast part (a linear model of price and dividend). A random process governs changes in dividends. At the start of the time period the current dividend is posted and observed by all agents. Each agent checks which of his predictors are “active”, that is, match the current state of the market. He then forecasts future price and dividend based on the most accurate of his active predictors and makes the appropriate bid or offer. The price is calculated by aggregating the agents’ demands and automatically clearing the market. At regular intervals, but asynchronously, agents engage in a learning process for updating their theories, where they discard unsuccessful strategies and evolve successful ones by a genetic algorithm, which mimics the process of natural selection by mutation and cross-over of the best performing rules.

**Fig. 1:**
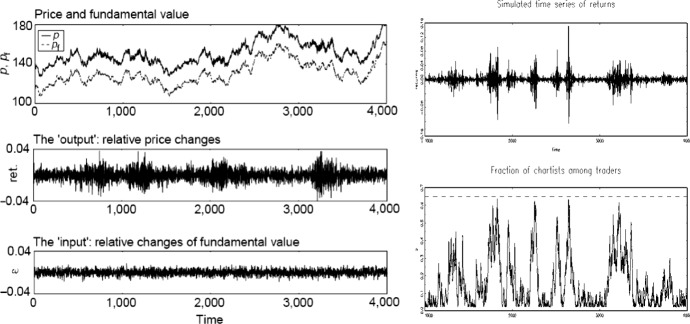
Left: the time series of prices closely tracks that of fundamentals (top); still, their difference shows volatility clustering (bottom) (Lux & Marchesi, 1999, p. 498). Right: price fluctuations (top) depend on the fraction of chartists in the market (bottom) (Lux & Marchesi, 2000, p. 689)

Simulations of the two models’ behaviour show that stylized facts obtain in both cases. In particular, simulations of the phase transition model show that the time series of the market price stays close to the time series of the fundamentals, in agreement with the hypothesis that price variations are unpredictable (Fig. 1, left, top). However, random changes in fundamentals (Fig. 1, left, bottom) do not result in similarly normally distributed returns, the time series of returns exhibiting a higher-than-normal frequency of extreme events and volatility clustering (Fig. 1, left, middle).^6^ For a wide range of parameter values, volatility bursts (Fig. 1, right, top) robustly depend on whether or not the proportion of chartists in the market exceeds a critical value (Fig. 1, right, bottom). Hence, the modellers conclude, volatility bursts are *explained* by the switching process (see Lux & Marchesi, 1999, p. 500, and Lux & Marchesi, 2000, p. 679).

**Fig. 2:**
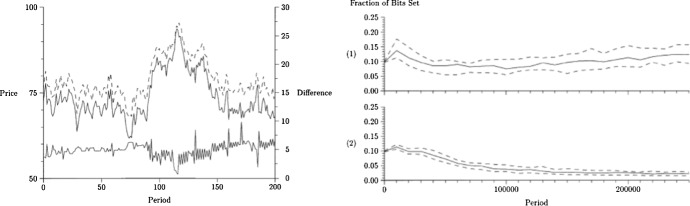
Left: the time series of prices closely tracks that of fundamentals (top); still, their difference (bottom) shows volatility clustering (LeBaron et al., 1999, p. 1500). Right: with fast learning, agents retain chartist strategies (top); conversely, with slow learning, agents discard them (bottom) (LeBaron et al., 1999, pp. 1506–1507)

Analogously, in the case of the evolutionary model, the price series tracks very closely the fundamental value series. Still, the time series of differences between prices and fundamental values shows the presence of both tranquil periods and severe fluctuations (Fig. 2, left). Moreover, the simulations reveal that different parameter values governing the learning speed result in different regimes. In slow learning conditions, the price series are indistinguishable from what should be produced in the case of homogeneous rational expectation equilibrium. In fast learning conditions, instead, stylized facts obtain. Statistical analyses show that with slow learning, traders learn that chartist bits are of no use and as time advances tend to eliminate them from their trading strategy (Fig. 2, bottom right); with fast learning, in contrast, chartist bits are useful, and thus their use does not decay as a result of learning (Fig. 2, top right). The modellers conclude that complex regimes arise in the absence of exogenous shocks *because of* the endogenous learning process.

It is important to emphasize that, in each case, the authors do not simply intend to explore the space of possible mechanisms responsible for the data but also to support a specific hypothesis in contradiction to EMH. The purported explanation of why stylized facts obtain is that they are the result of an endogenous self-reinforcing process and, ultimately, of the *heterogeneity* of the agents, in violation of REH (cf. Casini, 2014). In the authors’ own words:

Financial prices have been found to exhibit some universal characteristics that resemble the scaling laws characterizing physical systems in which large numbers of units interact. This raises the question of whether scaling in finance emerges in a similar way from the interactions of a large ensemble of market participants. However, such an explanation is in contradiction to the prevalent ‘efficient market hypothesis’ in economics, which assumes that the movements of financial prices are an immediate and unbiased reflection of incoming news about future earning prospects. Within this hypothesis, scaling in price changes would simply reflect similar scaling in the ‘input’ signals that influence them. Here we describe *a multi-agent model of financial markets which supports the idea that scaling arises from mutual interactions of participants*. Although the ‘news arrival process’ in our model lacks both power-law scaling and any temporal dependence in volatility, we find that it generates such behaviour as a result of interactions between agents (Lux & Marchesi, 1999, p. 498, our emphasis).

By now, enough statistical evidence has accumulated to question efficient-market theories and to show that the traders’ viewpoint cannot be entirely dismissed. As a result, the modern finance literature has been searching for alternative theories that can explain these market realities. (Arthur et al., 1997, Sect. 1) [...] We conjecture a simple evolutionary explanation. *Both in real markets and in our artificial market*, *agents are constantly exploring and testing new expectations*. Once in a while, randomly, more successful expectations will be discovered. Such expectations will change the market, and trigger further changes in expectations, so that small and large “avalanches” of change will cascade through the system. (Arthur et al., 1997, Sect. 5, our emphasis)

Both mechanisms rely on differences in individual behaviour—the emergence of chartist behaviour of some agents at some time, in one case, and the differences in the agents’ inductive and adaptive behaviour, in the other case. These differences trigger positive feedbacks inducing a self-reinforcing, endogenous process—by making switches between groups catalyse more switches, in one case, and by making successful use of chartist (or non-“rational”) strategies induce more and more agents to learn to behave in a chartist way, in the other case. This process generates the aforementioned stylized facts, namely fat tails, volatility persistence, and volatility clustering.^7^ The match between the model behaviour and the real data is interpreted by the authors as a confirmation of the heterogeneity hypothesis.

## The Model

### Informal Motivation

How do these models confirm the hypothesis that the agents’ heterogeneity is necessary to the stylized facts, given that many unrealistic components also enter the derivation of the stylized facts in each model? The answer, in short, is that the confirmation can depend on the robust dependence of the stylized facts on heterogeneity. Before presenting our formal reconstruction, let us provide an informal explication of this idea.

The two agent-based models at hand represent their target system, namely the financial market, in analogy with better-known source systems, in this case physical and biological systems. They are built on the assumption that the agents’ heterogeneity contributes to the stylized facts of finance. The way heterogeneity is represented, in turn, varies depending on the analogy that guides the model’s construction—the analogy between the market and a fluid undergoing phase transition in one case, and between the market and a population undergoing natural selection in the other case. It is *as if* agents switched or evolved and *as if*—as a result of this switch or evolution—transition cascades or evolution cascades obtained. The analogies have some intuitive plausibility. However, in light of the potential *dis*analogies involved in the models’ assumptions, the overall merit of these analogies is hard to evaluate *a priori*. It is here that RA comes into play.

In agent-based models, RA aims to assess the variability of the results depending on the model’s internal structure (Railsback & Grimm, 2011, pp. 302–306)—for instance, on the probability distributions used to set the parameters, on the functional forms used to relate the objects’ attributes, on the spatial/relational structure through which objects interact, on the objects’ invoking order and scheduling (on the last two aspects, see Axtell, 2001; Miller & Page, 2004). Rarely are empirical data available to calibrate all of these aspects of an agent-based model. This may generate scepticism as regards its results (see, e.g., Grüne-Yanoff, 2009b, p. 547). RA evaluates how the results vary when the non-calibrated components receive a different implementation. The more stable the results against such changes, the larger our confidence that the model explains the results.

In our chosen example, by varying assumptions for which no obvious justification is available, RA shows that the stylized facts are likely not an artifact of the model design, that is, an artifact of the possibly false idealizations used in the results’ derivation. To begin with, consider *the assumptions on the exact nature of heterogeneity*. The phase transition model assumes no learning (agents can only switch back and forth between groups), whereas the evolutionary model implements a simple form of learning (via the genetic algorithm). The phase transition model allows agents to interact (just as particles may bump into each other and change their state, so agents can meet and change their trading attitude), whereas the evolutionary model assumes no direct interaction (agents interact only indirectly, via the observation of prices, which are the aggregate result of all agents’ choices). Although agents are somewhat different from one another, both models involve misrepresentations of real-world heterogeneity.^8^ Since there is no intuitive reason for preferring one realization of heterogeneity to another, RA varies the respects in which heterogeneity is realized. Analogously, the *auxiliary assumptions* of the models are somewhat credible, but also involve misrepresentations of their real-world counterparts. At the same time, one has no reason to prefer one misrepresentation to another—for instance, to prefer a price determination determined by a Walrasian auctioneer, as in the phase transition model, to one governed by a market clearing condition, as in the evolutionary model. Therefore, RA varies the auxiliary assumptions of the models that cannot be calibrated. Since heterogeneity, albeit differently realized, is always part of the assumptions leading to the stylized facts, whereas the auxiliary assumptions vary from case to case, the hypothesis that heterogeneity (broadly construed) is necessary to the stylized facts gets more credible—or, in other words, *confirmed*—in light of the evidence, namely the results of the simulations. As it happens, in the case at hand not all model components are varied, and not all of them are varied to the same extent. For instance, both models assume that all agents have a simple portfolio consisting of one bond and one stock at all times. We will return to whether the lack of variation affects the possibility of confirmation by RA in Sect. 4.2.

In particular, we would want our account of confirmation by RA to rationalize the following intuitions: the more systems with heterogeneous agents reproduce the stylized facts, the larger the confirmation of the target hypothesis, and conversely, the more systems with homogeneous agents that comply with REH reproduce the stylized facts, the larger the *dis*confirmation. Moreover, the less dependent the auxiliaries of the former systems, the larger the confirmation, and conversely, the less dependent the auxiliaries of the latter systems, the larger the *dis*confirmation. Below, we show *how* and *when* one can rationalize these intuitions in Bayesian terms. Our choice is motivated by the goal of defending the confirmatory role of RA from an argument by Odenbaugh and Alexandrova (2011, p. 759) that hinges on the difficulty in using RA to “discharge” all of the auxiliaries employed in deriving a result of interest and on the lack of independence between the auxiliaries of the models obtained by RA. The analysis will be completed by an examination of cases of RA that go beyond the scope of Odenbaugh and Alexandrova’s own objections, namely the variation of the implementation of heterogeneity rather than the auxiliaries. In both cases, we will identify the conditions for RA to be confirmatory with respect to a target hypothesis.

In the literature, there are suggestions on using hypothetico-deductivism (H-D) to reconstruct RA (Levins, 1993, p. 553; Cartwright, 2009, p. 52; cf. Weisberg, 2006, p. 732; Kuorikoski et al., 2010, p. 560; for an elaboration, see Lehtinen, 2016, 2018). Very roughly, H-D is an account of scientific method, according to which scientists test scientific hypotheses by logically deducing observable consequences from them, and by falsifying the hypotheses if said consequences are not observed in reality, and corroborate, or confirm, them if the consequences are observed. There is a vast literature on the pros and cons of H-D. What is relevant for our purposes is that H-D does not allow one to explicitly formalize the notion of *degree* of confirmation. As such, it is not very suitable to address the critical target or our paper, namely the claim by Odenbaugh and Alexandrova that the difficulty in discharging all of the auxiliaries *weakens the dependence* of the phenomenon of interest on the hypothesis confirmed by RA. A counterargument must formally show what happens to the degree of confirmation of this hypothesis when not all auxiliaries may be discharged. Moreover, the second of Odenbaugh and Alexandrova’s objections is equally hard to address in the framework of H-D. Odenbaugh and Alexandrova argue that confirmation by RA hinges on the independence of the auxiliary assumptions of the models. What they mean is something stronger than non-identity: two auxiliaries may be dependent even if they are *not fully dependent*. Again, a suitable counterargument should show what happens when the idealization that the auxiliaries are independent is relaxed, and residual dependencies (which needn’t be as strong as full dependence) are introduced. To this end, it seems desirable to have a framework in which these dependencies may be formally represented.

To address Odenbaugh and Alexandrova’s objections, a natural option for us to explore is to adopt a framework, which is widely used to represent degrees of confirmation, namely Bayesian confirmation theory. Not only is the theory much more mainstream than H-D, nowadays, as a tool for reconstructing confirmation. There are also numerous suggestions in the literature to the point that Bayesianism may be fruitfully applied to the task of reconstructing RA (Schupbach, 2015, 2018; Stegenga & Menon 2017, ; see also Kuorikoski et al., 2010, p. 545). Another advantage of Bayesianism over H-D is that the former allows one to seamlessly integrate conflicting evidence, for instance, to integrate the existence of both models that confirm that heterogeneity is necessary to the stylized facts and of models that disconfirm this hypothesis.^9^

### Model Specification

In the following, we provide a Bayesian network model (adapted from Bovens & Hartmann, 2003, Sect. 4.4, using binary propositional variables) of how a target hypothesis is (dis)confirmed. Let us consider for concreteness the heterogeneity hypothesis of our example, which we denote by the variable *X*:

$$\begin{aligned} X={\left\{ \begin{array}{ll} x:&{}{\mathrm{In}\, \mathrm{reality},\, \mathrm{heterogeneous}\, \mathrm{agents}\, \mathrm{are}\, \mathrm{necessary}\, \mathrm{for}\, \mathrm{all}\, \mathrm{stylized}\, \mathrm{facts.}}\\ {\bar{x}}:&{}{\mathrm{In}\, \mathrm{reality},\, \mathrm{heterogeneous}\, \mathrm{agents}\, \mathrm{are}\, \mathrm{not}\, \mathrm{necessary}\, \mathrm{for}\, \mathrm{some}\, \mathrm{stylized}\, \mathrm{facts.}} \end{array}\right. } \end{aligned}$$

Note that the random walk of prices is a stylized fact that constitutes a benchmark for any acceptable model of asset pricing. This stylized fact is recovered by neoclassical models, where agents are homogeneous as entailed by REH. Henceforth, whenever we talk of “stylized facts” we mean those, which neoclassical economics has problems recovering, in particular the three stylized facts mentioned in Sect. 2.1—fat tails, volatility persistence, and volatility clustering—namely, those that, *in addition to* the random walk of prices, the aforementioned agent-based models but no neoclassical model can recover.

$X=x$ is logically equivalent to: stylized facts obtaining in reality entails that REH is false. In turn, the falsity of REH can be realized in many ways, because there are many ways for a group of agents to behave differently from one another, or heterogeneously. Thus, by “heterogeneous agents” we mean some form of heterogeneity or other, and not any specific type of heterogeneity. Conversely, $X={\bar{x}}$ means that at least one of those three stylized facts does not necessitate heterogeneity.

Prior to the development of heterogeneous models recovering stylized facts, it was somewhat plausible that $X=x$ is true (cf. quotes in Sect. 2.2 above). Hence, $0<P(x)<1$.

Following (Bovens & Hartmann, 2003) we insert intermediate variables between the hypothesis and the evidence to account for the fact that a hypothesis, in our case $X=x$, is confirmed via its testable consequences, in our case the existence of heterogeneous (HET) models that recover stylized facts. In general, models cannot be used to establish necessity claims, only the consistency of certain assumptions with the evidence. Moreover, any model only allows us to reason about those stylized facts it actually investigates. For a given HET model $M_i$ we hence define the intermediate variable as:

$$\begin{aligned} H_i={\left\{ \begin{array}{ll}h_i:&{}{\mathrm{In\, reality,\, heterogeneous\, agents\, as\, described\, by\, model}~ {M}_i~\mathrm{are\, consistent\, with\, all\, stylized\, facts}}\\ &{}{\mathrm{investigated\, by\, model} ~{M}_i.}\\ {\bar{h}}_i:&{}{\mathrm{In\, reality,\, heterogeneous\, agents\, as\, described\, by\, model}~ M_i~\mathrm{are\, inconsistent\, with\, some\, stylized}}\\ &{}{\mathrm{facts\, investigated\, by\, model}~ M_i.}\end{array}\right. } \end{aligned}$$

Confirmation of these intermediary variables, which are about specific kinds of heterogeneity being compatible with a number of stylized facts (fat tails, volatility clustering, and volatility persistence), raises the probability that heterogeneity of some kind or other is necessary to all stylized facts.

Since $H_i$ mentions a particular type of heterogeneity, the (non-)necessity of some kind of heterogeneity does not rule out nor does it imply the consistency of a particular kind of heterogeneity with the observation of stylized facts. Therefore, $P(h_i|x),P(h_i|{\bar{x}})\in (0,1)$.

Let us compare $P(h_i|x)$ to $P(h_i|{\bar{x}})$. In the former case it holds that heterogeneity is necessary for stylized facts in reality. Then some form of heterogeneity has to be consistent with the observed stylized facts. In the latter case it holds that, heterogeneity is not necessary for stylized facts in reality. It is hence possible that homogeneity is consistent with stylized facts in reality. The presence of *x* rather than ${\bar{x}}$ hence makes it more likely that some form of heterogeneity has to be consistent with the observed stylized facts. This in turn raises the probability that a particular heterogeneity assumption ($H_i$) is consistent with all stylized facts. Hence, $P(h_i|x)>P(h_i|{\bar{x}})$.^10^

Crucially, HET models rely on auxiliary assumptions, by which we mean all specifications of the model that do not concern the heterogeneity of the agents. To model this, we employ a variable $A_i$ with the following intended interpretation:

$$\begin{aligned} A_i={\left\{ \begin{array}{ll} a_i:&{}{\mathrm{The\, auxiliaries\, of\, model }\,M_i~\mathrm{describe\, a\, system,\, which\, is\, similar\, to\, reality}}.\\ {\bar{a}}_i:&{}{\mathrm{The\, auxiliaries\, of\, model }\,M_i~\mathrm{describe\, a\, system,\, which\, is\, not\, similar\, to\, reality.}} \end{array}\right. } \end{aligned}$$

Since $A_i=a_i$ is neither fully credible nor incredible, we have $0<P(a_i)<1$.^11^

Not every HET model is designed to recover all stylized facts.^12^ Our evidence variables $E_i$ are hence defined relative to a given subset of stylized facts (fat tails, volatility clustering, and volatility persistence):

$$\begin{aligned} E_i={\left\{ \begin{array}{ll}e_i:&{}{\mathrm{The\, HET\, model}~ M_i~\mathrm{recovers\, all\, the\, stylized\, facts\, investigated\, by\, model}~ M_i.}\\ {\bar{e}}_i:&{}{\mathrm{The\, HET\, model}~ M_i~\mathrm{does\, not\, recover\, some\, of\, the\, stylized\, facts\, investigated\, by\, model }~M_i}. \end{array}\right. } \end{aligned}$$

Notice that $H_i$ screens off $E_i$ from *X*: knowing the value of $H_i$ renders the value of *X* irrelevant to the probability of $E_i$. In the absence of knowledge of $A_i$, $H_i$ being true rather than false (i.e., the relevant heterogeneity is consistent with the stylized facts investigated by the model) renders $e_i$ more likely, $P(e_i|h_i)>P(e_i|{\bar{h}}_i)$. Since also $P(h_i|x)>P(h_i|{\bar{x}})$, it follows that $P(e_i|x_i)>P(e_i|{\bar{x}}_i)$. That is, these $e_i$ have Bayes factors greater than 1.^13^

The variables *X*, $H_i$ and $A_i$ all refer to facts *in reality*. To obtain evidence for the corresponding propositions, the result of the simulation, that is, the recovery of the stylized fact *in the model*, needs to be empirically validated. This empirical validation amounts to a non-trivial comparison of statistical features of simulation results and of real-world datasets, which themselves need to be empirically analysed.^14^ This is a non-trivial task for at least three reasons. First, the statistical assessment of the empirical properties of real-world datasets themselves is difficult, and not a yes-no matter (see, e.g., Cont, 2001). Second, there is still an open debate about how to adequately compare simulated data and real-world data, and different comparison methods may indicate that simulated data match real-world data to different degrees (for a review, see Fagiolo et al., 2019). Finally, there is an ongoing methodological debate on what counts as a successful replication in general, (see, e.g., Atmanspacher, 2016; Fletcher, 2021; Freedman, 2015; Rubin, 2021). So, while the result of the simulation contributes to the probability of $e_i$, it is not sufficient to determine the value of $E_i$ with certainty. Hence, $0<P(e_i)<1$.

Notice that the value of $E_i$ is not a direct consequence of the model’s assumptions, because knowledge of the values of $H_i$ and $A_i$ does not entail the match between the statistical properties of simulated data and real-world data. Thus, the conditional probabilities of $E_i$ are also non-extreme, $1>P(e_i|h_ia),P(e_i|h_i{\bar{a}}_i),P(e_i|{\bar{h}}_ia_i),P(e_i|{\bar{h}}_i{\bar{a}}_i)>0$. Moreover, whether or not the auxiliaries describe facts that are similar to reality (fixed truth value of $A_i$), the fact that the relevant heterogeneity is consistent with the stylized facts investigated by the model $M_i$ makes the model $M_i$ more likely to recover the investigated stylized facts, $P(e_i|h_ia_i)>P(e_i|{\bar{h}}_ia_i)$ and $P(e_i|h_i{\bar{a}}_i)>P(e_i|{\bar{h}}_i{\bar{a}}_i)$. Finally, whether or not the relevant heterogeneity is consistent with the stylized facts investigated by the model (fixed truth value of $H_i$), the fact that the auxiliaries describe facts that are similar to reality makes $e_i$ more probable, $P(e_i|h_ia_i)>P(e_i|h_i{\bar{a}}_i)$ and $P(e_i|{\bar{h}}_i a_i) >P(e_i|{\bar{h}}_i{\bar{a}}_i)$. In sum, we obtain:

$$\begin{aligned} 1\quad>\quad P(e_i|h_ia) \quad>\quad P(e_i|h_i{\bar{a}}_i),P(e_i|{\bar{h}}_ia_i) \quad> \quad P(e_i|{\bar{h}}_i{\bar{a}}_i) \quad >\quad 0. \end{aligned}$$

Note that $P(e_i|h_i{\bar{a}}_i),P(e_i|{\bar{h}}_i{\bar{a}}_i)>0$ guarantees the possibility of *ad hoc* HET models, which recover stylized facts thanks to auxiliaries explicitly designed to match the statistical features of observed data. As we explain in the next section, however, in that case the posterior of $X=x$ is approximately equal to its prior. That is, our Bayesian model does *not* rationalize, or justify, RA over *ad hoc* models, as it should be.

**Fig. 3:**
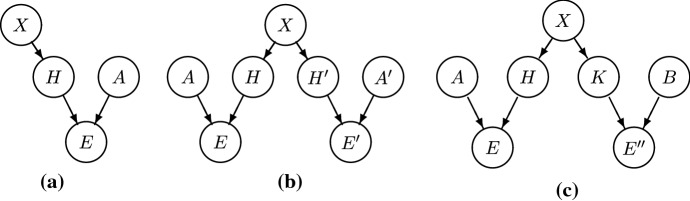
**a** A one-HET model; **b** a two-HET model; **c** a one-HET and one-HOM model

So far, our Bayesian model features edges from *X* pointing to the $H_i$, and edges from $H_i$ and $A_i$ pointing to $E_i$ (Fig. 3b). There are no edges between the $A_i$ and *X* and no edges between the $A_i$ and $H_j$ due to the lack of unconditional correlations between these variables. As regards the relation between the $A_i$, in Sect. 4.1 we assume that they are mutually independent. This assumption will be relaxed in Sect. 4.2.

Finally, to keep the mathematics tractable, we assume that the $H_i$ are also independent. This is a formal limitation of our model. If $X=x$ is true, then there is some (hypothetical) model which employs the sort of heterogeneous agents which are necessary for stylized facts in reality. Knowing that some other sorts of heterogeneity are not consistent with stylized facts clearly increases the probability that a so-far not-investigated sort of heterogeneity is consistent with stylized facts, $P(h_i|x{\bar{h}}_1\dots {\bar{h}}_{i-1})>P(h_i|x)$. There should hence be dependencies between the $H_i$ (cf. Claveau & Grenier, 2019). To alleviate the worry that the lack of dependencies drives our results below, we point to the fact that we establish strict inequalities, which continue to hold under small enough variations of our model due to the continuous dependence of posterior probabilities on prior probabilities, and that the great number of possible realizations of heterogeneity makes for weak dependencies between the $H_i$.

Our model can be extended to handle homogeneous (HOM) systems, namely systems with homogeneous agents that *dis*confirm $X=x$, if they recover stylized facts (Fig. 3c).

We again insert an intermediate variable, *K*, between the evidence from these models and *X*:Notice that, contrary to the case of HET systems, where we distinguish between different kinds of heterogeneity, compliance with REH entails *one* kind of homogeneity. The difference between HOM models depends on the different auxiliary assumptions they make. We hence chose to employ only a single binary propositional variable *K* to model homogeneity.

There are (still) economists who hold that $P(k)>0$, and so do we. Note that $X=x$ entails $K={\bar{k}}$, and so $P({\bar{k}}|x)=1$ and $P(k|x)=0$. If heterogeneity is not necessary for all stylized facts, then at least one stylized fact in addition to the random walk of prices is consistent with REH, namely $P(k|{\bar{x}})>0$. Furthermore, it is consistent with ${\bar{x}}$ that one stylized fact (e.g., volatility clustering) is consistent with the REH but another stylized fact (e.g., fat tails) is not. In this case, REH is not consistent with all stylized facts and so $P(k|{\bar{x}})<1$.

Similarly to the HET models, we use subscripted variables $E_{n+j}$ to denote the evidence from HOM models with the intended interpretation that $e_{n+j}$ indicates that HOM model $M_{n+j}$ recovers the stylized facts it investigates.

$$\begin{aligned} E_{n+j}={\left\{ \begin{array}{ll}e_i:&{}{\mathrm{The\, HOM\, model}\, M_{n+j}~\mathrm{recovers\, all\, the\, stylized\, facts\, investigated\, by\, model}~M_{n+j}.}\\ {\bar{e}}_{n+j}:&{}{\mathrm{The\, HOM\, model }\,M_{n+j} \,\mathrm{does\, not\, recover\, some\, stylized\, fact\, investigated\, by\, model }\,M_{n+j}.} \end{array}\right. } \end{aligned}$$

Finally, we use variables $B_j$ to denote the auxiliary assumptions of HOM models.

$$\begin{aligned} B_j={\left\{ \begin{array}{ll}b_j:&{}{\mathrm{The\, auxiliaries\, of\, model}~M_{n+j}~\mathrm{describe\, a\, system,\, which\, is\, similar\, to\, reality.}}\\ {\bar{b}}_j:&{}{\mathrm{The\, auxiliaries\, of\, model}~M_{n+j}~\mathrm{describe\, a\, system,\, which\, is\, not\, similar\, to\, reality.}} \end{array}\right. } \end{aligned}$$

Since $B_j=b_j$ is neither fully credible nor incredible, $0<P(b_j)<1$.

To illustrate, the match between the statistical features of simulated data and observed data, $e_{n+j}$, may confirm that rational expectations are consistent with all stylized facts, $K=k$, in the presence of such-and-such auxiliaries as described by $b_j$ (say, a sunspot assumption or a financial accelerator assumption, plus other assumptions). This, in turn, would cast doubt on the necessity of heterogeneous agents to stylized facts, $X=x$.

As was the case for $e_i$, the unconditional and conditional probabilities of $e_{n+j}$ are non-extreme, for the same reasons. $P(e_{n+j}|k{\bar{b}}_j),P(e_{n+j}|{\bar{k}}{\bar{b}}_j)>0$ guarantees the possibility of *ad hoc* HOM models. Being the models *ad hoc*, however, the prior of $B_j=b_j$ will be low, entailing no disconfirmation for $X=x$ (again, see next section for an explanation).

Note that $P(e_{n+j}|{\bar{k}})<P(e_{n+j}|k)$: a homogeneous model is more likely to recover stylized facts, if stylized facts can obtain in reality without giving up REH. It follows that $P(e_{n+j}|x)<P(e_{n+j}|{\bar{x}})$: *ceteris paribus*, a homogeneous model is less likely to recover stylized facts, if stylized facts cannot obtain in reality without heterogeneity. This means that HOM models recovering stylized facts disconfirm $X=x$ (see Theorem 1). As it happens, there are no (well-accepted) HOM models recovering stylized facts. The existence of HOM models not recovering stylized facts actually confirms $X=x$.

**Table 1:** Overview of employed variables, their intended interpretation and (conditional) probabilities

Variable	Intended interpretation	(Conditional) probabilities
*X*	Observed stylized facts entail the negation of REH	$0<P(x)<1$
$H_i$	Heterogeneity_i_ is consistent with some stylized facts	$0<P(h_i|{\bar{x}})<P(h_i|x)<1$
$A_i$	Auxiliary assumptions_i_ resemble reality	$0<P(a_i)<1$
$E_i$	HET model_i_ recovers some stylized facts	$1>P(e_i|h_ia)>P(e_i|h_i{\bar{a}}_i), P(e_i|{\bar{h}}_i{{a}}_i)$ $P(e_i|h_i{\bar{a}}_i),P(e_i|{\bar{h}}_ia_i) {>} P(e_i|{\bar{h}}_i{\bar{a}}_i) {>} 0$
*K*	REH is consistent with all stylized facts	$P(k|x)=0$ and $0<P(k|{\bar{x}})<1$
$B_j$	Auxiliary assumptions_j_ resemble reality	$0<P(b_j)<1$
$E_{n+j}$	HOM model_n+j_ recovers some stylized facts	$P(e_{n+j}|{\bar{k}})<P(e_{n+j}|k)$

## Analysis

Having motivated and detailed our model we now proceed to derive formal results. We begin by investigating independent auxiliary variables and later introduce dependencies between assumptions—be they auxiliary assumptions or assumptions o the implementation of heterogeneity.

### Independent Assumptions

For the Bayesian networks defined above with conditional probabilities as recalled in Table 1 we obtain our first result:^15^

#### Proposition 1

(One HET model, Fig. 3a) The confirmation function, $\Delta ^H(x)=P(x|e) - P(x)$, is positive.

While there is no RA due to the lack of variation across auxiliaries, there is some confirmation by a single HET model recovering stylized facts. Notice that the values of *P*(*e*|*ha*) and $P(e|{\bar{h}} a)$ matter significantly to the confirmation of $X=x$ only when the prior of $A=a$ is far from 0. The smaller the prior, the less they matter. In particular, if $A=a$ is very unlikely, then the amount of confirmation (mostly) depends on the quotient $\frac{P(e|h{\bar{a}})}{P(e|{\bar{h}}{\bar{a}})}$.

Moreover, if $A={\bar{a}}$ has been manufactured to reproduce the stylized facts independently of the other parts of the model (i.e., the model is *ad hoc*), $P(e|{\bar{a}} h)\approx P(e|{\bar{a}} {\bar{h}})\approx 1$, then that quotient ($\frac{P(e|h{\bar{a}})}{P(e|{\bar{h}}{\bar{a}})}$) is very close to 1. This quotient is a Bayes factor. Ceteris paribus, the further away it is from one, the greater the (dis)confirmation. As a result, the posterior of $X=x$ is very close to the prior of $X=x$, such that no significant confirmation for $X=x$ obtains.

Note that these considerations always apply to the posterior probability of $X=x$ given the evidence generated by certain models. The prior probability of $X=x$ is not affected by the existence of this evidence. In particular, it is not affected by the existence of *ad hoc* models; the existence of *ad hoc* models does not make the prior 0.

We may now similarly represent a second heterogeneous system, HET’, with three additional variables $H'$, $A'$ and $E'$ (Fig. 3b). Note that, although HET and HET’ are based on a heterogeneity assumption, this assumption is differently implemented by the two models. We represent the different implementations as different variables *H* and $H'$, which become independent given *X*. Similarly, the sets of auxiliaries in the two models, *A* and $A'$, are different. These variations make the addition of the second system, HET’, a case of RA relative to *X*. Under the assumptions that *A* and $A'$ are independent (to be relaxed in Sect. 4.2), and that both models recover stylized facts, one gets the first instance of confirmation of $X=x$ by RA:

#### Proposition 2

(Two HET models, Fig. 3b) The confirmation function, $\Delta ^{HH'}(x)=P(x|ee') - P(x|e)$, is positive.

What we said on the dependence of confirmation on non ad-hocness in the context of one model applies to the context of two models, too. If we are (almost) sure that $A_2={\bar{a}}_2$ and the model $M_2$ has been manufactured to reproduce the stylized facts, $E_2=e_2$ irrespective of whether $H_2$ is true or false ($\frac{P(e_2|h_2{\bar{a}}_2)}{P(e_2|{\bar{h}}_2{\bar{a}}_2)}\approx 1$), then $P(x|e_1 e_2)$ won’t be (noticeably) larger than $P(x|e_1)$. That is, RA is not useful for confirmation of $X=x$ if it relies on gathering evidence from novel but *ad hoc* models.

In a two-system model with one HET system and one HOM system (Fig. 3c), Proposition 2 may be reinterpreted as follows:

#### Proposition 3

(One HET and one HOM model, Fig. 3c) The confirmation function, $\Delta ^{HK}(x)=P(x|ee'')-P(x)$, is positive, if and only if *e* is better evidence for $X=x$ than $e''$ is evidence against $X=x$, $\frac{P(e|x)}{P(e|{\bar{x}})}>\frac{P(e''|{\bar{x}})}{P(e''|x)}$ or, equivalently, the body of evidence has a Bayes factor greater than 1, $\frac{P(ee''|x)}{P(ee''|{\bar{x}})}>1$.

That is, confirmation is positive, if and only if the evidence for $X=x$ (provided by the HET model) outweighs the evidence against $X=x$ (provided by the HOM model); which is equivalent to saying that the entire body of evidence supports $X=x$. This proposition shows that conflicting evidence is straightforwardly accounted for in a Bayesian framework. Of course, determining whether a given HET model confirms $X=x$ more than a given HOM model disconfirms $X=x$ is an empirical matter. On the one hand, HET models’ auxiliaries are more plausible and they clearly recover stylized facts, whereas HOM models make less plausible assumptions and/or less clearly recover stylized facts. On the other hand, the existence of a single HOM model recovering stylized facts may—in principle—rule out the necessity of heterogeneity, while any collection of HET models is only an indication of the necessity of heterogeneity. Notice that, if one is almost sure that the HOM model is *ad hoc*, confirmation does not depend much on the HOM model. Since the Bayes factor of the HOM model is close to 1, the degree of confirmation reduces to that resulting from the HET model alone, $P(x|ee'')-P(x)\approx P(x|e)-P(x)$.

**Fig. 4:**
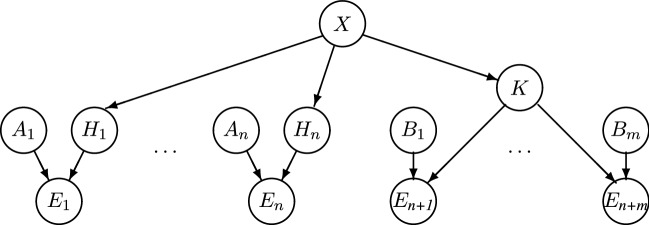
General *n*-HET and *m*-HOM model, under the assumption that $A_1,\ldots ,A_n,B_1,\ldots ,B_m$ are mutually independent

Let us now consider a general hypothetical case with an arbitrary number *n* of HET systems and an arbitrary number *m* of HOM systems (Fig. 4). We assume that different HET systems make different heterogeneity assumptions $H_i$ and that different $H_i$ are independent given *X*. By contrast, we assume that all HOM systems implement homogeneity in the same way, in line with REH, and we represent this fact by using a unique variable *K*.

The confirmation function, $\Delta ^{H_nK_m}(x)=P(x|e_1\ldots e_n e_{n+1}\ldots e_{n+m}) - P(x)$, has the following features: it monotonically increases in the number of HET models recovering stylized facts (*n*) and monotonically decreases in the number of HOM models recovering stylized facts (*m*). Assuming that these models provide non-vanishing evidence (the Bayes factors are bounded away from 1) and holding the HOM models fixed (*m* fixed), an ever greater number of HET models (*n* approaching infinity) raises the posterior probability of $X=x$ to approximating 1. Vice versa, holding the HET models fixed (*n* fixed), an ever greater number of HOM models (*m* approaching infinity) lowers the posterior probability of $X=x$ to approximating 0.

#### Theorem 1

(General *n*-HET and *m*-HOM, Fig. 4) The confirmation function $\Delta ^{H_nK_m}(x)=P(x|e_1\ldots e_n e_{n+1}\ldots e_{n+m}) - P(x)$ is strictly

1. increasing in *n*, and if there exists an $\epsilon >0$ such that for all *n* and all $1\le i\le n$ it holds that $\frac{P(e_i|x)}{P(e_i|{\bar{x}})}\ge 1+\epsilon$, then $\lim _{n\rightarrow \infty }P(x|e_1\ldots e_n e_{n+1}\ldots e_{n+m})=1$;
2. decreasing in *m*, and if there exists an $\epsilon '>0$ such that for all *m* and all $1\le j\le m$ it holds that $\frac{P(e_{n+j}|k)}{P(e_{n+j}|{\bar{k}})}\ge 1+\epsilon '$, then $\lim _{m\rightarrow \infty }P(x|e_1\ldots e_n e_{n+1}\ldots e_{n+m})=0$.

**Fig. 5:**
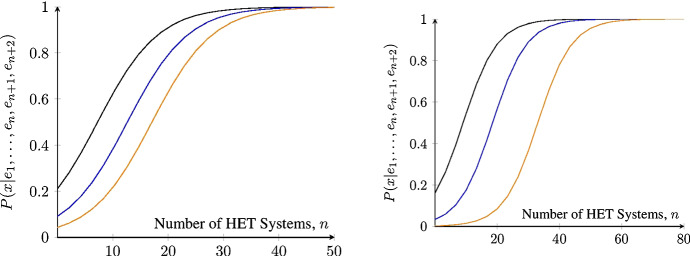
Posterior probability of $X=x$ according to Theorem 1 for varying number of HET systems, *n*. Left: the prior *P*(*x*) is set to 0.4 (black), 0.2 (blue), 0.1 (orange); fixed number of HOM systems, $m=2$. Right: number *m* of HOM systems is set to 1 (black), 3 (blue), 5 (orange); fixed prior of *x*, $P(x)=0.2$. To improve readability, *n* is displayed as a continuous variable. HET systems are less confirmatory than a HOM system throughout $\frac{P(e_i|x)}{P(e_i|{\bar{x}})}:=1.2\ll 4=:\frac{P(e_{n+j}|k)}{P(e_{n+j}|{\bar{k}})}$ for all $1\le i\le n$ and $1\le j\le m$ and $P(k|{\bar{x}})=0.1$

Let us now turn to some interesting consequences of these results in the presence of both HET and HOM systems (Fig. 5). For a large enough number of HET systems, *n*, the posterior probability of $X=x$ goes to 1, independently of the prior probability of $X=x$ (left) or the number of HOM systems (right). *Ceteris paribus*, the less confirmatory every single HET system, the lower the posterior probability of $X=x$; and the greater the number of HOM systems, the greater the number of HET systems required for the posterior probability of $X=x$ to become (almost) 1. Let us recall that a large posterior probability of $X=x$ under the assumption that stylized facts obtain in the real world is also a large probability that REH is false. Hence, if stylized facts do obtain in reality, then if many non-*ad-hoc* HET models with independent auxiliaries were to recover stylized facts and few non-*ad-hoc* HOM models with independent auxiliaries were to recover stylized facts, the posterior probability of $X=x$ would get very close to 1, which would bring the degree of belief in the REH close to 0. By contrast, an increasing number of HOM models recovering stylized facts would make us virtually sure that $K=k$ holds. Since $K=k$ cannot be true if $X=x$ is true, $P(k|x)=0$, the posterior probability of $X=x$ would get very close to 0, making us confident that $X=x$ is false.

**Fig. 6:**
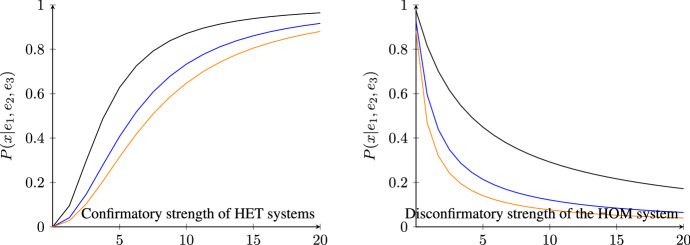
Posterior probability of $X=x$ according to Theorem 1 for two HET systems and one HOM system, fixed prior $P(x)=0.2$, and $P(k|{\bar{x}})=0.9$, against varying (dis)confirmatory strength. Confirmation strength is equal to $\frac{P(e_i|x)}{P(e_i|{\bar{x}})}$ for $i\in \{1,2\}$. Disconfirmation strength is equal to $\frac{P(e_3|{\bar{k}})}{P(e_3|k)}$. Left: disconfirmation strength is set to 4 (black), 10 (blue), and 15 (orange). Right: confirmation strength is set to $\sqrt{15}$ (black), $\sqrt{5}$ (blue), and $\sqrt{3}$ (orange)

The above results require a monotonic increase in the number *n* of HET systems. In practice, however, the number of systems studied by RA is always finite. As a result, the posterior of $X=x$ doesn’t reach extreme values, which would justify accepting or rejecting REH. In any given case, whether $X=x$ is confirmed, that is, whether $\Delta ^{H_n K_m}(x)$ is positive, depends on how strongly the HET models confirm $X=x$ and how strongly the HOM models disconfirm $X=x$. More concretely, let us consider what happens in a situation closer to our case study (Fig. 6), where one has evidence from *two* HET systems, namely the phase transition model and the evolutionary model, and *one* HOM system, say, one’s favourite DSGE model. If the evidence from HET systems is strong enough, few (two, here) HET systems suffice to significantly raise the posterior probability of $X=x$ (left). Similarly, if the evidence from the HOM systems is strong enough, few HOM systems suffice to lower the posterior probability of $X=x$ (right).

### Dependent Assumptions

#### General Considerations

The last section showed that RA can confirm a hypothesis under the assumption that the models’ auxiliaries are varied to the point that different sets of auxiliaries in different models become fully independent of one another. It is now time to relax that assumption, in order to address Odenbaugh and Alexandrova’s (2011) objections. To this end, let us first re-examine the objections.

**Fig. 7:**
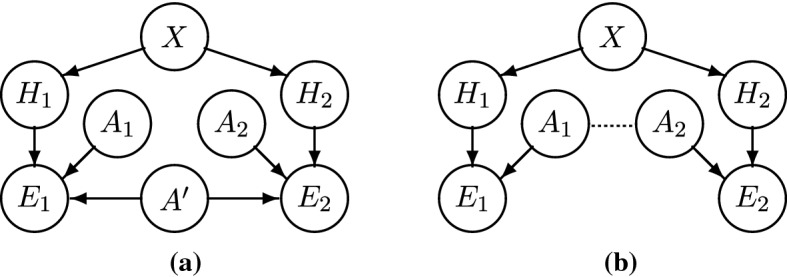
Two-system models with dependent auxiliaries: in (**a**), $A_1$ and $A_2$ denote fully independent sets of auxiliaries and $A'$ denotes a set of fully dependent auxiliaries; in (**b**), the dotted line denotes residual dependencies between sets of auxiliaries $A_1$ and $A_2$

To recall, the first objection is that “only some, not all, of the idealizations of models are discharged by robustness analysis”. In that case, so Odenbaugh and Alexandrova argue, confirmation by RA remains “empirically questionable and thus explanatorily weak”. In our framework, the undischarged idealizations Odenbaugh and Alexandrova talk about amount to non-varied auxiliaries (e.g., two-asset portfolios). For an illustration in the case of two systems, see Fig. 7a. There, some auxiliaries, $A_1$ and $A_2$, are fully independent, whereas the remaining auxiliaries, $A'$, are fully dependent (i.e., the two models have some but not all auxiliaries in common). For Odenbaugh and Alexandrova, this is problematic because, if the $A'$ are not varied by RA, one may not be able to tell whether the evidence depends on $A'$ rather than *X*.

A strengthened version of this first objection has been formulated by Lisciandra (2016), who points out that assumptions introduced for tractability reasons may not be relaxed, precisely because the problem at hand would not be tractable without them:

[...] in the case of complex models, whose components are in relation with each other partly to satisfy analytical requirements, it becomes more difficult to break them down into single units that can be exchanged with different ones. (83)

While we concede that this may be a problem in certain cases, we do not think it is a problem in general. For instance, in a neoclassical framework, analytic solvability requires one to impose that the system is at equilibrium. In the case of agent-based models, by contrast, analytic solvability is not a concern, because the models can only be studied by numerical simulations. So, the modeller can decide whether or not to impose equilibrium, based on different motivations. The phase transition model imposes an equilibrium condition in order to show that, even at equilibrium, the market can still be subject to volatility bursts. The evolutionary model, by contrast, imposes no such constraint, as it aims to show how equilibrium itself depends on learning speed. More generally, in the case of agent-based models, it is in principle possible to vary their auxiliaries, due to their modular character and there being no need of analytic solvability. In the words of the economists Fagiolo and Roventini:

[Agent-based models], contrary to neoclassical [models], do not impose any strong theoretical consistency requirements (e.g., equilibrium, representative individual assumptions, rational expectations). This is because they are not required ex-ante to be analytically solvable. [...] in absence of strong consistency conditions, assumptions can be replaced in a modular way, without impairing the analysis of the model. Indeed, in standard neoclassical models one cannot simply replace the optimization assumption with another one just because the model does not behave well, as that would possibly destroy its analytical solvability. This is not so in [agent-based models] (Fagiolo & Roventini, 2017, 5.30)

Of course, the problem remains that, even assuming that all auxiliaries are in principle dischargeable, in practice it may be very hard to discharge all of them. But is that necessarily bad for confirmation?

Stegenga and Menon (2017, p. 420) have already argued that variation of all assumptions is unnecessary to confirmation by RA. They claim that “robustness does not require the independence of all theoretical assumptions. Rather, it only requires independence of problematic or controversial auxiliary assumptions”. Stegenga and Menon argue that distinct consequences $E_1,\ldots ,E_n$ of a hypothesis of interest, say $X=x$, warrant a large confirmation (an “epistemic oomph”) *only if* *X* *d*-separates (i.e., renders independent) $E_1$,..., $E_n$.^16^ In turn, *d*-separation obtains, if and only if either there are no shared assumptions (i.e., the auxiliaries are fully independent) or they are fully credible (i.e., their prior probability is 1). As an illustration, consider the case where $n\!=\!2$ (Fig. 7a). If the prior probability of $A'=a'$ is not 1, *X* doesn’t *d*-separate *E* and $E'$. In practice, since the auxiliaries entering RA are *always uncertain*, such that their prior is definitely below 1; and since *there are*, typically, shared auxiliaries, only $\{X,A'\}$ *d*-separates the $E_i$; in which case, Stegenga and Menon claim, RA cannot provide large confirmation.

We agree with Stegenga and Menon that confirmation is guaranteed when *X* *d*-separates the evidence. This is in line with our argument: if $A_1$ and $A_2$ are independent, such that *X* *d*-separates *E* and $E'$, $\Delta ^{H_n}(x)$ is positive (Theorem 1). At the same time, Stegenga and Menon clearly intend independence as *necessary* to a robustness argument (cf. the above quote). On this, we disagree. Confirmation by RA obtains as long as *E* and $E'$ together confirm $X=x$ more than *E* or $E'$ alone. Since, intuitively, confirmation inversely co-varies with the dependence among the auxiliaries, confirmation may be *substantial* even if *X* fails to *d*-separate. To support this point, the question that we now want to address is: *When* and *how much* if at all, does RA confirm in the presence of dependencies? Answering this question will allow us to tackle at once both of Odenbaugh and Alexandrova’s (2011) objections.

Let us represent *non*-independence by a residual dependence between different sets of auxiliaries. Notice that the model in Fig. 7a is a special case of that in Fig. 7b, where only full dependence and independence are allowed, but no partial dependence. By contrast, the model in Fig. 7b is more general, as it captures not only full dependencies (i.e., identity) between auxiliaries but also partial dependencies (i.e., similarity). The latter suffices to leave different sets of auxiliaries correlated. To illustrate, not all of the auxiliaries in our case study change from one model to the other. Some assumptions are varied, for instance, those about interactions, about learning, and about equilibrium. Other assumptions stay the same, for instance, those about the agents’ portfolios. Others still are only “partly” varied. For instance, a Walrasian auctioneer (phase transition model) and an automatic market clearing (evolutionary model) are different ways to implement a centralized price determination mechanism, yet they are more similar to one another than to a *de*centralized price determination mechanism. Analogously, the assumptions that fundamentals change at random (phase transition model) and that dividends change at random (evolutionary model) are different ways to implement exogenous shocks, but are more similar to one another than assuming that these shocks have a systematic component due to a common cause, say, a sunspot. Any of these similarities entails a—more or less strong—partial dependence.

Recall that the second objection is that “[r]obustness analysis crucially depends on showing that the assumptions of different models are independent of one another” but this condition is typically *not* satisfied. The first objection, then, may be interpreted as a special case of the second: undischarged idealizations entail the identity, and thus the *lack of variation*, of some auxiliaries, entailing in turn a residual dependence.

We shall now present results supporting *three conclusions*, which show that the above objections are invalid (1 and 2), but at the same time emphasize that the usefulness of RA for confirmation is sensitive to conditions, which require careful scrutiny (3):

- Conclusion 1: Discharging of auxiliaries is unnecessary to a confirmation increase from further evidence.
- Conclusion 2: Discharging of auxiliaries is unnecessary to maximal confirmation, that is, to $P(x|\text {Evidence})=1$.
- Conclusion 3: Sometimes replication is more confirmatory than variation with respect to auxiliaries as well as heterogeneity assumptions.

#### Formal Analysis and Interpretation of Results

**Conclusion 1.** There are many ways in which a number of auxiliaries can be dependent on each other. The extreme case obtains, if all auxiliaries are fully dependent on each other, i.e., there is only one single auxiliary assumption. Graphically speaking, there is only one single auxiliary variable, $A'$, in Fig. 7, which is the parent of two or more evidential variables. Assuming that every evidential variables *E* has a distinct variable *H* as a parent, Osimani and Landes (forthcoming, Sect. 3.4, Scenario 2) show that $P(x|e_1\dots e_{n+1})-P(x|e_1\dots e_n)$ increases in certain cases. Assuming that all models make the same auxiliary assumptions, the more HET models recover stylized facts, the greater the confirmation for $X=x$.

Following (Bovens & Hartmann, 2003, Eq. 4.13), Osimani and Landes (forthcoming, Sect. 3.4) however also discover cases in which a further HET model does *decrease* confirmation.^17^ In such cases, learning that a second model recovers stylized facts means that we also learn about the truth value of $A'$. If the second HET model strongly boosts our belief in a truth value $A'=a'$ such that it is $A'=a'$ which is responsible for the recovering of stylized facts, i.e., it is not $X=x$ which drives the recovering of stylized facts, then the second model recovering stylized facts *decreases* the confirmation of $X=x$ – for suitable parameters, because $A'=a'$ is the main driver of the recovery of the stylized facts and not $X=x$.

Upshot 1: Independence of auxiliaries is not *necessary* for a confirmation increase. Nor is it sufficient: there are cases in which confirmation *decreases*.

**Conclusion 2.** This leaves open the question of when confirmation can become maximal (a posterior probability of $X=x$ equal to 1 obtains).

We investigate two cases: (2a) As above: only one auxiliary variable $A'$, many HET models $E_1,\dots ,E_n$ and every HET model has its own distinct heterogeneity variable $H_i$. (2b) We add a distinct variables $A_i$ representing auxiliary assumptions specific to individual HET models.

(2a) In order to make calculations tractable, we make the ceteris paribus assumptions that the conditional probabilities $P(e_i|xa'),P(e_i|{\bar{x}},a'),P(e_i|x,{\bar{a}}'),$

$P(e_i|{\bar{x}},{\bar{a}}'),P(h_i|x),P(h_i|{\bar{x}})$ do not depend on *i*. We hence unambiguously write $P(e|xa'), P(h|x)$, and so on.

##### Proposition 4

(Convergence Result) $P(e|x,a')>P(e|{\bar{x}},a')$ and $P(e|x,{\bar{a}}')>P(e|{\bar{x}},{\bar{a}}')$ jointly entail for all $P(x)\in (0,1), P(a')\in (0,1)$ that

$$\begin{aligned} \lim _{n\rightarrow \infty }P(x|e_1,\dots ,e_n)=1 . \end{aligned}$$

In words: if HET models recovering stylized facts are evidence for $X=x$ for all fixed truth values of $A'$, then the posterior of $X=x$ converges to 1 with an increasing number of *independent* HET models (independent given *X* and $A'$).

Upshot 2a: Different auxiliaries are not necessary for maximal confirmation. Every new HET model increases confirmation—under reasonable assumptions. There is no need to vary auxiliaries to obtain a posterior of *x* to approximating 1, all one needs is sufficiently many HET models which are evidence for $X=x$.

(2b) Different HET models now make different auxiliary assumptions, but some auxiliary assumptions are also shared. We model this as follows: The shared assumptions are represented by a variable $A'$. The auxiliaries particular to every model are represented by $A_i$.^18^

Again, in order to make calculations tractable, we make the ceteris paribus assumptions that the conditional probabilities $P(e_i|xaa'),P(e_i|xa{\bar{a}}'),P(e_i|{\bar{x}},aa'), P(e_i|{\bar{x}},a {\bar{a}}'),P(e_i|x,{\bar{a}}a')$,$P(e_i|x,{\bar{a}}{\bar{a}}'),P(e_i|{\bar{x}},{\bar{a}}a'),P(e_i|{\bar{x}},{\bar{a}}{\bar{a}}')$ do not depend on *i*. We hence unambiguously write $P(e|xaa'),P(e|x{\bar{a}}a'), P(h|x)$, and so on.

##### Proposition 5

(Convergence Result—One shared auxiliary assumption $A'$ + model-specific auxiliaries $A_i$, Fig. 7a for $n=2$). $P(e|x,a',a)> P(e|{\bar{x}},a',a)$, $P(e|x,{\bar{a}}',a)> P(e|{\bar{x}},{\bar{a}}',a)$, $P(e|x,a',{\bar{a}})> P(e|{\bar{x}},a',{\bar{a}})$ and $P(e|x,{\bar{a}}',{\bar{a}})> P(e|{\bar{x}},{\bar{a}}',{\bar{a}})$ jointly entail for all $P(x)\in (0,1), P(a)\in (0,1)$ that

$$\begin{aligned} \lim _{n\rightarrow \infty }P(x|e_1,\dots ,e_n)=1 . \end{aligned}$$

The condition says: The presence of $X=x$, rather than $X={\bar{x}}$, makes the HET models more likely to recover stylized facts – for all fixed possible values of the shared and the model-specific auxiliary assumptions (fixed truth values of $A'$ and $A_i$).

Upshot 2b: Multiple auxiliaries are not necessary for maximal confirmation *even for the more realistic assumption of some auxiliaries assumptions being shared*. Every new HET model increases confirmation—under reasonable assumptions. There is no need to vary auxiliaries to obtain a posterior of $X=x$ approximating 1; all one needs is sufficiently many HET models which are evidence for $X=x$.

**Conclusion 3.** We have seen that RA via variation of auxiliary assumptions can increase confirmation—even to the maximum. We have also seen that variation is not always confirmatory. This raises the question of whether it is more confirmatory to *not vary* assumptions in order to boost the posterior probability of $X=x$.

The literature on the Variety of Evidence Thesis (VET) has a direct bearing on the issue. The thesis says that varied evidence for a hypothesis confirms it more strongly than less varied evidence, ceteris paribus. This thesis has recently come under heavy scrutiny (Au, 2021; Bovens & Hartmann, 2002, 2003; Cartwright, 2021; Claveau, 2011, 2013; Claveau & Grenier, 2019; Couch, forthcoming; Hartmann & Bovens, 2001; Landes, 2020a, b, 2021; Osimani & Landes, forthcoming; Schupbach, 2015). A number of surprising results showed that there are cases in which less varied evidence, ceteris paribus, confirms more strongly than more varied evidence. Typically, these results are due to the fact that repeatedly applying the same experimental methodology not only tests the hypothesis of interest but *also* tests the methodology employed. Repeated tests employing the same methodology may greatly enhance the assessed quality of the methodology. In turn, this enhancement boosts our confidence in the hypothesis of interest being true. In *some cases*, this boost of confidence outweighs the confidence boost one would have obtained from testing with varied methodologies, ceteris paribus. The VET hence fails in such cases.

**Fig. 8:**
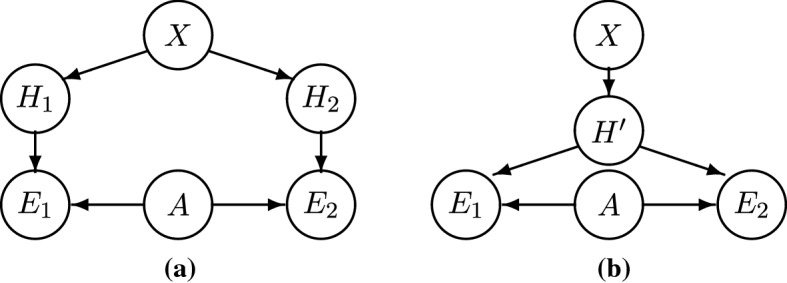
Variation vs replication of heterogeneity assumptions. In (**a**), $H_1$ and $H_2$ denote fully independent implementations of heterogeneity; in (**b**) $H'$ denotes the same implementation. Here, $E_1$ and $E_2$ are reinterpreted relative to the match between model behaviour and reality in, respectively, the first half and the second half of a series of observations. In (**a**), the first half of the series is matched with data generated by model $M_1$ and the second half is matched with data generated by model $M_2$; in (**b**), the whole series of observations is used to validate the same model $M'$

In what follows, we bring to bear this discussion to that on the confirmatory role of RA by construing the works on the VET as an approach to RA. To this end, we interpret evidential variety as varied auxiliaries or varied heterogeneity assumptions and the VET itself as a thesis about the confirmatory value of RA. First, we present existing results from this discussion which are relevant to our response to Odenbaugh and Alexandrova, where we show when employing the same auxiliaries grants more confirmation than varying them (3a and 3b). Next, we build on existing literature by providing a novel result concerning the variation of heterogeneity assumptions (see Fig. 8), where we show when employing the same heterogeneity assumption can grant more confirmation than varying it (3c). Here we anticipate the relevant upshots for our purposes. (A more accurate discussion of Conclusion 3 requires introducing further technical details, which we leave to the next, separate section. The interested reader may find there a formal statement of these upshots and a precise explication of them.)

**Fig. 9:**
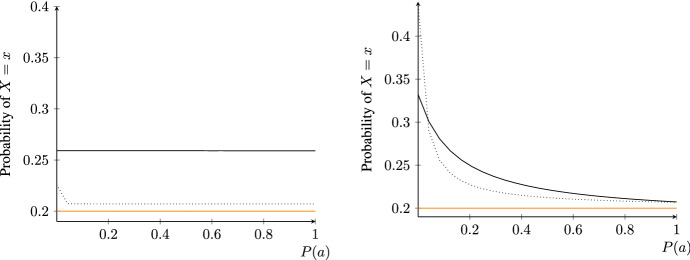
Posterior probabilities of $X=x$ of varied (solid) and not varied (dashed) heterogeneity assumptions as in Proposition 8 for a prior of $P(x)=20\%$ (orange) depending on the prior of *A*, *P*(*a*). Whenever the solid line is above the dotted line variation outperforms replication, otherwise replication outperforms variation. Since all posteriors are above the orange line, positive confirmation always obtains. Left (parameter values $P(x)=0.2, P(h|x)=0.35,P(h|{\bar{x}})=0.25,P(e|ha)=0.63,P(e|{\bar{h}}a)=0.02,P(e|h{\bar{a}})=0.51,P(e|{\bar{h}}{\bar{a}})=0.01)$): variation always confirms more than replication. Right (parameter values $P(x)=0.2, P(h|x)=0.2,P(h|{\bar{x}})=0.1,P(e|ha)=0.39,P(e|{\bar{h}}a)=0.32,P(e|h{\bar{a}})=0.34,P(e|{\bar{h}}{\bar{a}})=0.01)$): replication confirms more than variation only for small *P*(*a*)

- Upshot 3a: Gradually increasing the independence of the auxiliaries $A_i$ is not required for a confirmation increase. Sometimes, gradually increasing the independence *decreases* confirmation (See Proposition 6 for a precise statement.)
- Upshot 3b: Independence of auxiliaries $A_i$ is not required for a confirmation increase. Sometimes, full dependence of auxiliaries is more confirmatory than full independence of auxiliaries. (See Proposition 7 for a precise statement.)
- Upshot 3c: Full independence of heterogeneity assumptions $H_i$ is not required for a confirmation increase. Sometimes, full dependence of heterogeneity assumptions is more confirmatory than full independence of heterogeneity assumptions (See Proposition 8 for a precise statement.) Figure 9 serves as an illustration.

#### Conclusion 3 in More Detail

Two different types of models investigated in the recent literature on the VET are relevant to our current purposes, namely models where the dependence between auxiliaries is a *gradual* notion, and models where auxiliary variables are *either fully dependent* (i.e., there is only one single auxiliary variable which is a parent of all evidential variables) *or fully independent*.

As concerns the first kind, Landes (2021) shows that confirmation may increase or decrease by gradually increasing independence of HET models, by an investigation of ${{\,\mathrm{sign}\,}}(\frac{\partial }{\partial \uplambda }P(x|ee'))$, where $\uplambda$ is a parameter representing the degree of (in)dependence between auxiliaries. Consider the case depicted in Fig. 7b with two auxiliary variables which are dependent to a degree. Landes (2021) puts forward an explication of a degree of independence in terms of a real-valued single parameter $\uplambda \in [0,1]$. For $\uplambda =0$ full dependence obtains and for $\uplambda =1$ there is full independence. Making the, by now usual, ceteris paribus assumptions that the index *i* can be ignored, and letting $\rho :=P(a),1-\epsilon _+ =P(e|h,a),\epsilon _- :=P(e|{\bar{h}},a),\alpha :=P(e|h,{\bar{a}}),\gamma :=P(e|{\bar{h}},{\bar{a}}),p:=P(h|x),q:=P(h|{\bar{x}})$, the main result for our purposes is:

##### Proposition 6

(Proposition 9 of Landes 2021) For all $P(a),\alpha ,\gamma ,\epsilon _- ,\epsilon _+ ,P(x)\in (0,1)$ it holds that

$$\begin{aligned}&{{\,\mathrm{sign}\,}}\Bigl (\frac{\partial }{\partial \uplambda }P(x|e_1e_2)\Bigr )={{\,\mathrm{sign}\,}}\Bigl (\Bigl (\alpha \epsilon _- -\gamma (1-\epsilon _+ )\Bigr )\cdot \\&\quad \left( P(a)\cdot [\epsilon _- [(1-\epsilon _+ )-\alpha ]+(1-\epsilon _+ )[\epsilon _- -\gamma ]]+(1-P(a)) \right. \\&\quad \left. \cdot [\gamma ((1-\epsilon _+ )-\alpha )+\alpha (\epsilon _- -\gamma )]\right) \Bigr ) . \end{aligned}$$

Upshot 3a: Gradually increasing the independence of the auxiliaries is not required for a confirmation increase. Sometimes, gradually increasing the independence *decreases* confirmation.^19^

The interpretation of this result will be discussed after presenting one more result, which contrasts full dependence and full independence, and which uses a model of the second kind. As an illustration of this contrast, consider Fig. 7a. The model obtained by deleting the node $A'$ represents the *variation* case in which each evidential variable has a distinct auxiliary variable as a parent. The model obtained by deleting nodes $A_1$ and $A_2$ represents the *replication* case in which both evidential variables have the same auxiliary variable as a parent. Making the standard ceteris paribus assumptions, Osimani and Landes (forthcoming) identify the conditions under which replication is more confirmatory than variation:

##### Proposition 7

(Theorem 1 in Osimani and Landes, forthcoming) For all $p=P(h|x)\in (0,1)$, $q=P(h|{\bar{x}})\in (0,p)$, $\rho =P(A=a)\in (0,1)$, $\epsilon _+ ,\epsilon _- \in (0,1)$, $\alpha \in (1-\epsilon _+ ,1)$ and $\gamma \in (\epsilon _- ,1)$ replication is more confirmatory than variation, if and only if

$$\begin{aligned} 0<\gamma _2\le \gamma \le \frac{\epsilon _- }{1-\epsilon _+ }\cdot \alpha <1, \end{aligned}$$

where $\gamma _2$ is the following parameter

$$\begin{aligned} \gamma _2:=\frac{{\epsilon _- }\cdot [2\rho (1-\epsilon _+ )+\alpha (1-2\rho )]}{(2\rho -1)(1-\epsilon _+ )+2\alpha (1-\rho )} . \end{aligned}$$

This result can do with some explanation. Why is it that replication can be more confirmatory than variation?

Recall that in our model, $A=a$ increases the probability of a HET model recovering stylized facts: $P(e|xa)>P(e|x{\bar{a}})$ and $P(e|{\bar{x}}a)>P(e|{\bar{x}}{\bar{a}})$. This means that multiple HET models recovering stylized facts increases our belief in $A=a$. Furthermore, in order to apply the above result we need to identify *P*(*e*|*ha*) with $\alpha$, $P(e|{\bar{h}} a)$ with $\gamma$, $1-\epsilon _+$ with $P(e|h{\bar{a}})$ and $\epsilon _-$ with $P(e|{\bar{h}}{\bar{a}})$.

Next, note that replication trumps variation (in the parameter range specified in Proposition 7), if and only if the following two (logically equivalent) inequalities hold

$$\begin{aligned} \frac{\alpha }{1-\epsilon _+ }=\frac{P(e|ha)}{P(e|h\bar{a})}>\frac{P(e|\bar{h}a)}{P(e|\bar{h}\bar{a})}=\frac{\gamma }{\epsilon _- }\\ \frac{\alpha }{\gamma }=\frac{P(e|ha)}{P(e|\bar{h}a)}>\frac{P(e|h\bar{a})}{P(e|\bar{h}\bar{a})}=\frac{1-\epsilon _+ }{\epsilon _- }. \end{aligned}$$

Since the $H_i$ are probabilistic consequences of *X*, comparative confirmation of $H_i=h_i$ obtains, if and only if it obtains for $X=x$. We hence phrase our explanation below in terms of confirmation of $X=x$.

The second inequality says that $A=a$ is more confirmatory for $X=x$ than $A={\bar{a}}$ is. So, in order to strongly increase our belief in $X=x$ we should strongly increase our belief in $A=a$. Now, consider the case in which one HET model recovers stylized facts: the second instance of a HET model recovering stylized facts is judged to be more likely to originate from $A=a$, if there is a single *A* variable rather than two distinct *A* variables. So, adding an auxiliary variable leads to a smaller belief in $A= a$ than going for a replication approach. Hence, the replication strategy confirms the auxiliary hypothesis more strongly than the variety strategy. Hence, replication confirms $X=x$ more strongly than variation.

Let us now illustrate the case at the opposite end of the spectrum, where there is full dependence among auxiliaries (which is a special case of the two inequalities in Proposition 7 not being both satisfied). Let us consider an instance in which $A={\bar{a}}$ turns the HET model in a “randomizer”, which recovers stylized facts (or not) independently from reality, $P(e|x{\bar{a}})=P(e|{\bar{x}}{\bar{a}})$. So, for $A=a$ for which $P(e|xa)>P(e|{\bar{x}}a)$, the recovery of stylized facts provides some evidence for $X=x$, we thus test *A* over and over again to best confirm that $A=a$. In this case, replication trumps variation.

Upshot 3b: Independence of auxiliaries is not required for a confirmation increase. Sometimes, full dependence of auxiliaries is more confirmatory than full independence of auxiliaries.

When only two models are considered, full independence and full dependence in Proposition 7 are special cases of gradual variations of (in)dependence of auxiliaries as described in Proposition 6. In fact, Proposition 6 studies intermediate (in)dependencies, which are convex combinations of these two extremes.^20^ As one moves from one extreme to the other, confirmation is either always positive or always negative—which explains Upshot 3a.

So far, we have only considered variations in the auxiliary assumptions (3a and 3b). However, the heterogeneity assumption can be varied, too (3c). The question arises whether the latter kind of variation, too, boosts confirmation. Such a problem case can be visualised by contrasting Fig. 8a, where the distinction between $H_1$ and $H_2$ represents variation, and Fig. 8b, where $H_1$ and $H_2$ are merged into a single variable $H'$ to represent replication. The question of whether variation is more confirmatory than replication with respect to testable consequences of the hypothesis of interested has been investigated by Claveau and Grenier (2019) and Landes (2020b). However, neither of these results fit our model specifications, some of the variables in Claveau and Grenier (2019) are ternary and the conditional probabilities used differ from ours, while Landes (2020b) does not consider auxiliary assumptions.

To investigate this new case, we hence compare confirmation from two HET models recovering stylized facts which make the same set of auxiliary assumptions. In the replication case (*R*) both models make the same heterogeneity assumption, while in the variation case (*V*) the two models make different heterogeneity assumptions. Making the usual ceteris paribus assumptions we find (proof in Appendix 2):

##### Proposition 8

(Variation vs. Replication of Heterogeneity Assumptions) For all $p=P(h|x)\in (0,1)$, $q=P(h|{\bar{x}})\in (0,p)$, $\rho =P(a)\in (0,1)$, $\epsilon _+ ,\epsilon _- ,\alpha ,\gamma \in (0,1)$ it holds that

$$\begin{aligned} {{\,\mathrm{sign}\,}}(&P_R(x|e_1,e_2)-P_V(x|e_1,e_2)) \\ =&-{{\,\mathrm{sign}\,}}\Bigl (\rho ^2\cdot ((1-\epsilon _+ )-\epsilon _- )^2\cdot \Bigl (\frac{(1-\epsilon _+ )^2}{\epsilon _- ^2}-\frac{{\bar{p}}{\bar{q}}}{pq}\Bigr )\\&\quad +{\bar{\rho }}^2\cdot (\alpha -\gamma )^2\cdot \Bigl (\frac{\alpha ^2}{\gamma ^2} -\frac{{\bar{p}}{\bar{q}}}{pq}\Bigr )\\&+\rho {\bar{\rho }}\cdot \Bigl (\frac{(1-\epsilon _+ )^2\gamma ^2+\epsilon _- ^2\alpha ^2+2[-\epsilon _- \alpha -(1-\epsilon _+ )\gamma +(1-\epsilon _+ )\alpha ]\cdot (1-\epsilon _+ )\alpha }{(1-\epsilon _+ )^2\gamma ^2+\epsilon _- ^2\alpha ^2+2[-\epsilon _- \alpha -(1-\epsilon _+ )\gamma +\epsilon _- \gamma ]\cdot \epsilon _- \gamma }\\&\quad -\frac{{\bar{p}}{\bar{q}}}{p q}\Bigr )\Bigr ) . \end{aligned}$$

In the borderline case of $\rho =P(a)=1$, the intuitive result (variation trumps replication) obtains, if and only if $\left( \frac{P(e|ha)}{P(e|{\bar{h}} a)}\right) ^2>\frac{P({\bar{h}}|{\bar{x}})P({\bar{h}}|x)}{P(h|{\bar{x}})P(h|x)}$, the same expression as in Landes (2020b). $P(a)=1$ means that *A* is not really a variable since we are sure about its value. We can hence remove the variable from consideration and are hence back in the exact situation as in (Landes, 2020b). Conversely, the borderline case ${\bar{\rho }}=1$ produces the intuitive result, if and only if $\left( \frac{P(e|h{\bar{a}})}{P(e|{\bar{h}}{\bar{a}})}\right) ^2>\frac{P({\bar{h}}|{\bar{x}})P({\bar{h}}|x)}{P(h|{\bar{x}})P(h|x)}$. This is the analogue of the first case with *a* and ${\bar{a}}$ permuted.

In the more interesting case of $0<\rho =P(a)<1$, there is no such simple expression which tells us whether variation trumps replication or vice versa.

Upshot 3c: Full independence of heterogeneity assumptions is not required for a confirmation increase. Sometimes, full dependence of heterogeneity assumptions is more confirmatory than full independence of heterogeneity assumptions.

#### Summary

Our Bayesian model shows that not only are independence and *d*-separation *unnecessary* to confirmation, despite claims to the opposite by respectively Odenbaugh and Alexandrova (2011) and Stegenga and Menon (2017); under some conditions, dependence actually bestows *more* confirmation than independence. Clearly, under those conditions, trying to build models with as diverse as possible assumptions is not going to boost confirmation of the hypothesis under investigation. In those cases, the use of RA for confirmatory purposes is not vindicated, and replication strategies are more confirmatory than variation strategies. At the same time, our model shows that, in other cases, the opposite is true. Given suitable probability judgments, RA can boost confirmation and the use of RA for confirmatory purposes is vindicated. RA may raise the posterior probability of that hypothesis, at times even to the maximum.

Clearly, our model is motivated by a specific case study. Some of our results only concern pairs of models. Moreover, we do not mention alternative model relations between evidence and hypothesis, as discussed for instance by Wheeler and Scheines (2013). But already in this small set of circumstances we can see that there is no simple one-to-one relation between confirmation increase and (in)dependencies. That is, there is no obvious simple rule, which tells us whether RA is successful with increasing independence, or whether variation trumps replication. The expressions characterizing the cases discussed in this section are sensitive to many parameters and are not likely to be intuited prior to formal analysis. If confirmation hinges on unjustified probability assignments, we agree with Odenbaugh and Alexandrova that it remains “empirically questionable and thus explanatorily weak”. However, theoreticians are sometimes in the position to form plausible probability judgments given their knowledge of the subject matter they investigate. Relative to such judgments, there will be a whole spectrum of clear-cut cases, where RA can confirm hypotheses of interest.

It goes beyond the scope of this paper to discuss which proportion of the theoreticians’ probability judgments fall into the region in which our Bayesian model vindicates the use of RA for confirmatory purposes, irrespective of the hypothesis to be confirmed and the particularities of the case. In our case study from macroeconomics, we speculate that the persistence of the conflicting views on REH could be reconstructed as fundamental disagreement on the right probability assignments in the communities favouring REH and opposing it. We leave to future (empirical) work the survey and the comparison of such assignments.

## Conclusion

One can find the following two widespread views in the literature. On the one hand, there is the view that the production and exploration of “minimal”, highly idealized models cannot lead to the confirmation of empirical hypotheses. On the other hand, there is the view that performing robustness analysis (RA) on models does not allow them to confirm empirical hypotheses, for two reasons. Confirmation (allegedly) depends on, first, discharging all of the idealizations, namely on varying all of the auxiliary assumptions used in the construction of the models, and second, on rendering the models’ assumptions independent, namely on eliminating the residual dependencies between the auxiliaries being varied. In this paper, by reference to a case study from agent-based computational macroeconomics, we argued against such views. Minimal models *can* confirm—in a Bayesian sense—*in virtue of* RA.

To recall our main results, we showed that under the assumption of independence of the auxiliaries there is Bayesian confirmation (Proposition 2), which increases with accumulating supporting evidence (Theorem 1). Moreover, even if the independence assumption is relaxed and residual dependencies among the auxiliary and/or heterogeneity assumptions are allowed, RA can still confirm. Confirmation of hypotheses can even achieve the maximal attainable value, such that their posterior probability converges to 1 (Propositions 4 and 5). At the same time, we also drew on the literature on the so-called Variety of Evidence Thesis, which is concerned with the confirmatory role of replication vs variation, to identify the conditions under which (in)dependence is conducive to confirmation (Propositions 6 to 8). We showed that there are cases where, if the assumptions of different models are not independent from one another, RA is less confirmatory than replication. This is not to say that RA is not confirmatory *tout court*, however. In other cases, in fact, more independent assumptions make RA more confirmatory than replication.

In sum, contrary to a popular opinion, we concluded that RA can be key to hypothesis confirmation. This casts new light on the confirmatory power of minimal models. Even when the collection of novel empirical evidence and the direct inspection of real mechanisms is infeasible or impossible, the “mere” production and exploration of the behaviour of models can lend support to an empirical hypothesis. A benefit of our result is that it provides a formal framework, in which one can investigate the justificatory conditions for the practice of those large communities of scientists (not only economists but also, say, theoretical physicists or archaeologists), who for lack of better alternatives are forced to explore surrogates of reality (models) rather than reality itself in order to provide actual explanations of phenomena.

## Appendix 1: Independent Auxiliaries

Proposition 3 (One HET and one HOM model) The confirmation function, $\Delta ^{HK}(x)=P(x|ee'')-P(x)$, is positive, if and only if *e* is better evidence for $X=x$ than $e''$ is evidence against $X=x$, $\frac{P(e|x)}{P(e|{\bar{x}})}>\frac{P(e''|{\bar{x}})}{P(e''|x)}$ or, equivalently, the body of evidence has a Bayes factor greater than one, $\frac{P(ee''|x)}{P(ee''|{\bar{x}})}>1$.

### Proof

Simply note that

$$\square $$

Theorem 1 (General n-HET and m-HOM model; cf. Fig. 4) The confirmation function $\Delta ^{H_nK_m}(x)=P(x|e_1\ldots e_n e_{n+1}\ldots e_{n+m}) - P(x)$ is strictly

1. increasing in *n*, and if there exists an $\epsilon >0$ such that for all *n* and all $1\le i\le n$ it holds that $\frac{P(e_i|x)}{P(e_i|{\bar{x}})}\ge 1+\epsilon$, then $\lim _{n\rightarrow \infty }P(x|e_1\ldots e_n e_{n+1}\ldots e_{n+m})=1$;
2. decreasing in *m*, and if there exists an $\epsilon '>0$ such that for all *m* and all $1\le j\le m$ it holds that $\frac{P(e_{n+j}|k)}{P(e_{n+j}|{\bar{k}})}\ge 1+\epsilon '$, then $\lim _{m\rightarrow \infty }P(x|e_1\ldots e_n e_{n+1}\ldots e_{n+m})=0$.

### Proof

Using that $P({\bar{k}}|x)=1$ and $P(k|x)=0$ we obtain

$$\begin{aligned} P(x|&e_1\ldots e_n e_{n+1}\ldots e_{n+m})=\frac{P(xe_1\ldots e_n e_{n+1}\ldots e_{n+m})}{P(xe_1\ldots e_n e_{n+1}\ldots e_{n+m})+P({\bar{x}}e_1\ldots e_n e_{n+1}\ldots e_{n+m})}\\ =&\frac{1}{1+\frac{P({\bar{x}})}{P(x)}\frac{P(e_1\ldots e_n|{\bar{x}})P(e_{n+1}\ldots e_{n+m}|{\bar{x}})}{P(e_1\ldots e_n|x)P(e_{n+1}\ldots e_{n+m}|x)}}\\ =&\frac{1}{1+\frac{P({\bar{x}})}{P(x)}\cdot \prod _{i=1}^n\frac{P(e_i|{\bar{x}})}{P(e_i|x)}\cdot \frac{P(k|{\bar{x}})\prod _{j=1}^mP(e_{n+j}|k)+P({\bar{k}}|{\bar{x}})\prod _{j=1}^mP(e_{n+j}|{\bar{k}})}{P(k|x)\prod _{j=1}^mP(e_{n+j}|k)+P({\bar{k}}|x)\prod _{j=1}^mP(e_{n+j}|{\bar{k}})}}\\ =&\frac{1}{1+\frac{P({\bar{x}})}{P(x)}\cdot \prod _{i=1}^n\frac{P(e_i|{\bar{x}})}{P(e_i|x)}\cdot [P(k|{\bar{x}})\prod _{j=1}^m\frac{P(e_{n+j}|k)}{P(e_{n+j}|{\bar{k}})}+P({\bar{k}}|{\bar{x}})]} . \end{aligned}$$

Inspecting the latter, we derive both claims concerning the increasing number of HET systems, *n*.

Claim 2 follows once we see that $P(e_{n+j}|{\bar{x}})>P(e_{n+j}|x)(1+\epsilon ')$ is logically equivalent to $P(e_{n+j}|k)>P(e_{n+j}|{\bar{k}})\delta$, where $\delta$ is greater than but arbitrarily close to one and is some constant that does not depend on *n*, *j*, *m* (in our case $\delta =1+\frac{\epsilon '}{P(k|{\bar{x}})}$). Using that $P(k|{\bar{x}})>0$ we now show this logical equivalence

$$\begin{aligned}&P(e_{n+j}|{\bar{x}})>P(e_{n+j}|x)(1+\epsilon ')\\&\Longleftrightarrow P(e_{n+j}|k)P(k|{\bar{x}})+P(e_{n+j}|{\bar{k}})P({\bar{k}}|{\bar{x}})> [P(e_{n+j}|k)P(k|x)\\&+P(e_{n+j}|{\bar{k}})P({\bar{k}}|x)](1+\epsilon ')\\&\Longleftrightarrow P(e_{n+j}|k)P(k|{\bar{x}})+P(e_{n+j}|{\bar{k}})(1-P(k|{\bar{x}}))> P(e_{n+j}|{\bar{k}})(1+\epsilon ')\\&\Longleftrightarrow P(e_{n+j}|k)P(k|{\bar{x}})> P(e_{n+j}|{\bar{k}})(P(k|{\bar{x}})+\epsilon ')\\&\Longleftrightarrow P(e_{n+j}|k) > P(e_{n+j}|{\bar{k}})(1+\frac{\epsilon '}{P(k|{\bar{x}})}) . \end{aligned}$$

Claim 1 simply follows by letting $\epsilon '=0$ in which case $\delta =1$. $\square$

## Appendix 2: Dependent Auxiliaries

Proposition 4 (Convergence Result) $P(e|x,a')>P(e|{\bar{x}},a')$ and $P(e|x,{\bar{a}}')>P(e|{\bar{x}},{\bar{a}}')$ jointly entail for all $P(x)\in (0,1), P(a')\in (0,1)$ that

$$\begin{aligned} \lim _{n\rightarrow \infty }P(x|e_1,\dots ,e_n)=1 . \end{aligned}$$

### Proof

$$\begin{aligned} P&(x|e_1,\dots ,e_n)=\frac{P(x,e_1,\dots ,e_n)}{P(e_1,\dots ,e_n)}\\&=\frac{P(x,e_1,\dots ,e_n,a')+P(x,e_1,\dots ,e_n,{\bar{a}}')}{P(x,e_1,\dots ,e_n,a')+P(x,e_1,\dots ,e_n,{\bar{a}}')+P({\bar{x}},e_1,\dots ,e_n,a')+P({\bar{x}},e_1,\dots ,e_n,{\bar{a}}')}\\&=\frac{1}{1+\frac{P({\bar{x}},e_1,\dots ,e_n,a')+P({\bar{x}},e_1,\dots ,e_n,{\bar{a}}')}{P(x,e_1,\dots ,e_n,a')+P(x,e_1,\dots ,e_n,{\bar{a}}')}}\\&=\frac{1}{1+\frac{P({\bar{x}})P(a')P(e_1,\dots ,e_n|{\bar{x}},a')+P({\bar{x}})P({\bar{a}}')P(e_1,\dots ,e_n|{\bar{x}},{\bar{a}}')}{P(x)P(a')P(e_1,\dots ,e_n|x,a')+P(x)P({\bar{a}}')P(e_1,\dots ,e_n|x,{\bar{a}}')}}\\&=\frac{1}{1+\frac{P({\bar{x}})}{P(x)}\cdot \frac{P(a')\prod _{i=1}^nP(e_i|{\bar{x}},a)+P({\bar{a}}')\prod _{i=1}^nP(e_i|{\bar{x}},{\bar{a}}')}{P(a')\prod _{i=1}^nP(e_i|x,a')+P({\bar{a}}')\prod _{i=1}^nP(e_i|x,{\bar{a}}')}}\\&=\frac{1}{1+\frac{P({\bar{x}})}{P(x)}\cdot \frac{P(a')P(e|{\bar{x}},a')^n+P({\bar{a}}')P(e|{\bar{x}},{\bar{a}}')^n}{P(a')P(e|x,a')^n+P({\bar{a}}')P(e|x,{\bar{a}}')^n}} . \end{aligned}$$

Let $\epsilon >0$ be fixed and given. Then the following two inequalities are logically equivalent

$$\begin{aligned}&\frac{P(a')P(e|{\bar{x}},'a)^n+P({\bar{a}}')P(e|{\bar{x}},{\bar{a}}')^n}{P(a)P(e|x,a')^n +P({\bar{a}}')P(e|x,{\bar{a}}')^n}<\epsilon \\&\quad P(a')(P(e|{\bar{x}},a')^n-\epsilon P(e| x a')^n)+P({\bar{a}}')(P(e|{\bar{x}}, {\bar{a}}')^n-\epsilon P(e|x {\bar{a}}')^n) <0 . \end{aligned}$$

If $P(e|x,a')>P(e|{\bar{x}},a')$ and $P(e|x,{\bar{a}}')>P(e|{\bar{x}},{\bar{a}}')$, this holds for all large enough *n*. In turn, the ratio following $\frac{P({\bar{x}})}{P(x)}$ converges to 0. The entire expression hence converges to 1. $\square$

Proposition 5 (Convergence Result – One shared auxiliary assumption $A'$ + model-specific auxiliaries $A_i$, Fig. 7a for $n=2$). $P(e|x,a',a)> P(e|{\bar{x}},a',a)$, $P(e|x,{\bar{a}}',a)> P(e|{\bar{x}},{\bar{a}}',a)$, $P(e|x,a',{\bar{a}})> P(e|{\bar{x}},a',{\bar{a}})$ and $P(e|x,{\bar{a}}',{\bar{a}})> P(e|{\bar{x}},{\bar{a}}',{\bar{a}})$ jointly entail for all $P(x)\in (0,1), P(a)\in (0,1)$ that

$$\begin{aligned} \lim _{n\rightarrow \infty }P(x|e_1,\dots ,e_n)=1 . \end{aligned}$$

### Proof

To simplify notation we denote negation by ^0^ while ^1^ denotes identity. We calculate and obtain:

Let $\epsilon >0$ be fixed and given. Then the following inequalities are logically equivalent

$$\begin{aligned}&\frac{P(a')[P(a)P(e|a{\bar{x}}a')+P({\bar{a}})P(e|{\bar{a}}{\bar{x}}a')]^n+P({\bar{a}}')[P(a)P(e|a{\bar{x}}{\bar{a}}')+P({\bar{a}})P(e|{\bar{a}}{\bar{x}}{\bar{a}}')]^n}{P(a')[P(a)P(e|axa')+P({\bar{a}})P(e|{\bar{a}}xa')]^n+P({\bar{a}}')[P(a)P(e|ax{\bar{a}}')+P({\bar{a}})P(e|{\bar{a}}x{\bar{a}}')]^n}<\epsilon \\&P(a')\cdot \Bigl ([P(a)P(e|a{\bar{x}}a')+P({\bar{a}})P(e|{\bar{a}}{\bar{x}}a')]^n\\&\quad -\epsilon [P(a)P(e|axa')+P({\bar{a}})P(e|{\bar{a}}xa')]^n\Bigr )\\&+P({\bar{a}}')\cdot \Bigl ([P(a)P(e|a{\bar{x}}{\bar{a}}')+P({\bar{a}})P(e|{\bar{a}}{\bar{x}}{\bar{a}}')]^n\\&\quad -\epsilon [P(a)P(e|ax{\bar{a}}')+P({\bar{a}})P(e|{\bar{a}}x{\bar{a}}')]^n\Bigr )<0 . \end{aligned}$$

Since $P(e|x,a',a)> P(e|{\bar{x}},a',a)$, $P(e|x,{\bar{a}}',a)> P(e|{\bar{x}},{\bar{a}}',a)$, $P(e|x,a',{\bar{a}}_i)> P(e|{\bar{x}},a',{\bar{a}})$ and $P(e|x,{\bar{a}}',{\bar{a}})> P(e|{\bar{x}},{\bar{a}}',{\bar{a}})$, the last inequality is true for all large enough *n*.

Hence, the ratio following $\frac{P({\bar{x}})}{P(x)}$ converges to 0. $P(x|e_1,\dots ,e_n)$ hence converges to 1. $\square$

Proposition 8 (Variation vs. Replication of Heterogeneity Assumptions) For all $p=P(h|x)\in (0,1)$, $q=P(h|{\bar{x}})\in (0,p)$, $\rho =P(a)\in (0,1)$, $\epsilon _+ ,\epsilon _- ,\alpha ,\gamma \in (0,1)$ it holds that

$$\begin{aligned} {{\,\mathrm{sign}\,}}(&P_R(x|e_1,e_2)-P_V(x|e_1,e_2)) \\ =&-{{\,\mathrm{sign}\,}}\Bigl (\rho ^2\cdot ((1-\epsilon _+ )-\epsilon _- )^2\cdot \Bigl (\frac{(1-\epsilon _+ )^2}{\epsilon _- ^2}-\frac{{\bar{p}}{\bar{q}}}{pq}\Bigr ) +{\bar{\rho }}^2\cdot (\alpha -\gamma )^2\cdot \Bigl (\frac{\alpha ^2}{\gamma ^2} -\frac{{\bar{p}}{\bar{q}}}{pq}\Bigr )\\&+\rho {\bar{\rho }}\cdot \Bigl (\frac{(1-\epsilon _+ )^2\gamma ^2+\epsilon _- ^2\alpha ^2+2[-\epsilon _- \alpha -(1-\epsilon _+ )\gamma +(1-\epsilon _+ )\alpha ]\cdot (1-\epsilon _+ )\alpha }{(1-\epsilon _+ )^2\gamma ^2+\epsilon _- ^2\alpha ^2+2[-\epsilon _- \alpha -(1-\epsilon _+ )\gamma +\epsilon _- \gamma ]\cdot \epsilon _- \gamma } -\frac{{\bar{p}}{\bar{q}}}{p q}\Bigr )\Bigr ) . \end{aligned}$$

### Proof

$$\begin{aligned} P_R(x|e_1,e_2)=&\frac{P(x,e_1,e_2)}{P(e_1,e_2)}=\frac{\sum _{H}\sum _{A}P(e_1,e_2,x,H,A)}{\sum _{X}\sum _{H}\sum _{A}P(e_1,e_2,X,H,A)}\\ =&\frac{P(x)[p\rho P(e|h,a)^2 + p{\bar{\rho }} P(e|h,{\bar{a}})^2+{\bar{p}}\rho P(e|{\bar{h}},a)^2+{\bar{p}}{\bar{\rho }} P(e|{\bar{h}},{\bar{a}})^2]}{P(x)[p\rho P(e|h,a)^2 + p{\bar{\rho }} P(e|h,{\bar{a}})^2+{\bar{p}}\rho P(e|{\bar{h}},a)^2+{\bar{p}}{\bar{\rho }} P(e|{\bar{h}},{\bar{a}})^2]+}\\&\;\frac{}{P({\bar{x}})[q\rho P(e|h,a)^2 + q{\bar{\rho }} P(e|h,{\bar{a}})^2+{\bar{q}}\rho P(e|{\bar{h}},a)^2+{\bar{q}}{\bar{\rho }} P(e|{\bar{h}},{\bar{a}})^2]}\\ =&\frac{P(x)[p\rho (1-\epsilon _+ )^2 + p{\bar{\rho }} \alpha ^2+{\bar{p}}\rho \epsilon _- ^2+{\bar{p}}{\bar{\rho }} \gamma ^2]}{P(x)[p\rho (1-\epsilon _+ )^2 + p{\bar{\rho }} \alpha ^2+{\bar{p}}\rho \epsilon _- ^2+{\bar{p}}{\bar{\rho }} \gamma ^2]+}\\&\;\frac{}{P({\bar{x}})[q\rho (1-\epsilon _+ )^2 + q{\bar{\rho }} \alpha ^2+{\bar{q}}\rho \epsilon _- ^2+{\bar{q}}{\bar{\rho }} \gamma ^2]}\\ =&\frac{1}{1+\frac{P({\bar{x}})[q\rho (1-\epsilon _+ )^2 + q{\bar{\rho }} \alpha ^2+{\bar{q}}\rho \epsilon _- ^2+{\bar{q}}{\bar{\rho }} \gamma ^2]}{P(x)[p\rho (1-\epsilon _+ )^2 + p{\bar{\rho }} \alpha ^2+{\bar{p}}\rho \epsilon _- ^2+{\bar{p}}{\bar{\rho }} \gamma ^2]}}\\ \\ P_V(x|e_1,e_2)=&\frac{P(x,e_1,e_2)}{P(e_1,e_2)}=\frac{\sum _{H_1}\sum _{H_2}\sum _{A}P(e_1,e_2,x,H_1,H_2,A)}{\sum _{X}\sum _{H_1}\sum _{H_2}\sum _{A}P(e_1,e_2,X,H_1,H_2,A)}\\ =&\frac{1}{1+\frac{P({\bar{x}})\sum _{H_1}\sum _{H_2}\sum _{A}P(E,H_1,H_2,A|{\bar{x}})}{P(x)\sum _{H_1}\sum _{H_2}\sum _{A}P(E,H_1,H_2,A|x)}}\\ =&\frac{1}{1+\frac{P({\bar{x}})[\rho (q(1-\epsilon _+ )+{\bar{q}}\epsilon _- )^2+{\bar{\rho }}(q\alpha +{\bar{q}}\gamma )^2]}{P(x)[\rho (p(1-\epsilon _+ )+{\bar{p}}\epsilon _- )^2+{\bar{\rho }}(p\alpha +{\bar{p}}\gamma )^2]}} . \end{aligned}$$

To see which posterior is greater, we consider ${{\,\mathrm{sign}\,}}(P_R(x|E)-P_V(x|E))$. To simplify notation we put $r:=\frac{1-\epsilon _+ }{\epsilon _- }$ and $s:=\frac{\alpha }{\gamma }$

$$\begin{aligned}&[\rho (q(1-\epsilon _+ )+{\bar{q}}\epsilon _- )^2+{\bar{\rho }}(q\alpha +{\bar{q}}\gamma )^2][p\rho (1-\epsilon _+ )^2 + p{\bar{\rho }} \alpha ^2+{\bar{p}}\rho \epsilon _- ^2+{\bar{p}}{\bar{\rho }} \gamma ^2] \\ -&[\rho (p(1-\epsilon _+ )+{\bar{p}}\epsilon _- )^2+{\bar{\rho }}(p\alpha +{\bar{p}}\gamma )^2][q\rho (1-\epsilon _+ )^2 + q{\bar{\rho }} \alpha ^2+{\bar{q}}\rho \epsilon _- ^2+{\bar{q}}{\bar{\rho }} \gamma ^2]\\ =&\rho ^2[(q(1-\epsilon _+ )+{\bar{q}}\epsilon _- )^2(p(1-\epsilon _+ )^2+{\bar{p}}\epsilon _- ^2)-(p(1-\epsilon _+ )+{\bar{p}}\epsilon _- )^2\\&\quad (q(1-\epsilon _+ )^2+{\bar{q}} \epsilon _- ^2)]\\&+{\bar{\rho }}^2[(q\alpha +{\bar{q}}\gamma )^2(p\alpha ^2+{\bar{p}}\gamma ^2)-(p\alpha +{\bar{p}}\gamma )^2(q\alpha ^2+{\bar{q}}\gamma ^2)]\\&+\rho {\bar{\rho }}[(q(1-\epsilon _+ )+{\bar{q}}\epsilon _- )^2(p\alpha ^2+{\bar{p}}\gamma ^2)+(q\alpha +{\bar{q}}\gamma )^2(p(1-\epsilon _+ )^2+{\bar{p}}\epsilon _- ^2)\\&\;\;-(p(1-\epsilon _+ )+{\bar{p}}\epsilon _- )^2(q\alpha ^2+{\bar{q}}\gamma ^2)-(p\alpha +{\bar{p}}\gamma )^2(q(1-\epsilon _+ )^2+{\bar{q}} \epsilon _- ^2)]\\ =&\rho ^2[(1-\epsilon _+ )^4pq(q-p)+\epsilon _- ^4{\bar{p}}{\bar{q}}({\bar{q}}-{\bar{p}})\\ {}&+(1-\epsilon _+ )^2\epsilon _- ^2(q^2{\bar{p}}+{\bar{q}}^2p-p^2{\bar{q}}-{\bar{p}}^2q)\\&+2q{\bar{q}}(1-\epsilon _+ )\epsilon _- (p(1-\epsilon _+ )^2+{\bar{p}}\epsilon _- ^2)-2p{\bar{p}}(1-\epsilon _+ )\epsilon _- (q(1-\epsilon _+ )^2+{\bar{q}} \epsilon _- ^2)]\\&+{\bar{\rho }}^2[\alpha ^4pq(q-p)+\gamma ^4{\bar{p}}{\bar{q}}({\bar{q}}-{\bar{p}})+\alpha ^2\gamma ^2(q^2{\bar{p}}+{\bar{q}}^2p-p^2{\bar{q}}-{\bar{p}}^2{\bar{q}})\\&\;\;\;\;\;+2q{\bar{q}}\alpha \gamma (p\alpha ^2+{\bar{p}}\gamma ^2)-2p{\bar{p}}\alpha \gamma (q\alpha ^2+{\bar{q}}\gamma ^2)]\\&+\rho {\bar{\rho }}[2(1-\epsilon _+ )^2\alpha ^2(q^2p-p^2q) + 2\epsilon _- ^2\gamma ^2({\bar{q}}^2{\bar{p}}-{\bar{p}}^2{\bar{q}})\\&\;\;\;\;+(1-\epsilon _+ )^2\gamma ^2(q^2{\bar{p}}+{\bar{q}}^2p-p^2{\bar{q}}-{\bar{p}}^2q)+ \epsilon _- ^2\alpha ^2({\bar{q}}^2 p+q^2{\bar{p}}-{\bar{p}}^2q-p^2{\bar{q}})\\&\;\;\;\;+2q{\bar{q}}(1-\epsilon _+ )\epsilon _- (p\alpha ^2+{\bar{p}}\gamma ^2)+2q{\bar{q}}\alpha \gamma (p(1-\epsilon _+ )^2+{\bar{p}}\epsilon _- ^2)\\&\;\;\;\;-2p{\bar{p}}(1-\epsilon _+ )\epsilon _- (q\alpha ^2+{\bar{q}}\gamma ^2)+2p{\bar{p}}\alpha \gamma (q(1-\epsilon _+ )^2+{\bar{q}}\epsilon _- ^2)]\\ =&\rho ^2[(1-\epsilon _+ )^4pq(q-p)+\epsilon _- ^4{\bar{p}}{\bar{q}}(p-q)+(1-\epsilon _+ )^2\epsilon _- ^2(q{\bar{p}}(q-{\bar{p}})\\ {}&+{\bar{q}}p({\bar{q}}-p))\\&\;\;+2qp(1-\epsilon _+ )^3\epsilon _- ({\bar{q}}-{\bar{p}})+2{\bar{p}}{\bar{q}}(1-\epsilon _+ )\epsilon _- ^3(q-p)]\\&+{\bar{\rho }}^2[\alpha ^4pq(q-p)+\gamma ^4{\bar{p}}{\bar{q}}(p-q)+\alpha ^2\gamma ^2(q{\bar{p}}(q-{\bar{p}})+{\bar{q}}p({\bar{q}}-p))\\&\;\;\;\;\;+2qp\alpha ^3\gamma ({\bar{q}}-{\bar{p}})+2{\bar{p}}{\bar{q}}\alpha \gamma ^3(q-p)]\\&+\rho {\bar{\rho }}[2(1-\epsilon _+ )^2\alpha ^2pq(q-p) + 2\epsilon _- ^2\gamma ^2{\bar{p}}{\bar{q}}({\bar{q}}-{\bar{p}})\\&\;\;\;\;\;\;\;\; +(1-\epsilon _+ )^2\gamma ^2(q^2{\bar{p}}+{\bar{q}}^2p-p^2{\bar{q}}-{\bar{p}}^2q)\\ {}&+ \epsilon _- ^2\alpha ^2({\bar{q}}^2p+q^2{\bar{p}}-{\bar{p}}^2q-p^2{\bar{q}})\\&\;\;\;\;+2pq\alpha ^2(1-\epsilon _+ ) \epsilon _- ({\bar{q}}-{\bar{p}})+2{\bar{p}}{\bar{q}}\gamma ^2(1-\epsilon _+ )\epsilon _- (q-p)\\&\;\;\;\;+2pq\alpha \gamma (1-\epsilon _+ )^2({\bar{q}}-{\bar{p}})+ 2{\bar{p}}{\bar{q}}\alpha \gamma \epsilon _- ^2(q-p)]\\ =&\rho ^2[(1-\epsilon _+ )^4pq(q-p)-\epsilon _- ^4{\bar{p}}{\bar{q}}(q-p)+(1-\epsilon _+ )^2\epsilon _- ^2((q-pq)\\&\quad (p+q-1)+(p-pq)(1-p-q))\\&\;\;+2qp(1-\epsilon _+ )^3\epsilon _- (p-q)+2{\bar{p}}{\bar{q}}(1-\epsilon _+ )\epsilon _- ^3(q-p)]\\&+{\bar{\rho }}^2[\alpha ^4pq(q-p)+\gamma ^4{\bar{p}}{\bar{q}}(p-q)+\alpha ^2\gamma ^2((q-pq)(p+q-1)\\&\quad +(p-pq)(1-p-q))\\&\;\;\;\;\;+2qp\alpha ^3\gamma (p-q)+2{\bar{p}}{\bar{q}}\alpha \gamma ^3(q-p)]\\&+\rho {\bar{\rho }}[2(1-\epsilon _+ )^2\alpha ^2pq(q-p) + 2\gamma ^2\epsilon _- ^2(p-q){\bar{p}}{\bar{q}}\\&\;\;\;\;\;\;\;\;+ \gamma ^2(1-\epsilon _+ )^2 (q^2-pq^2 + p-2pq+pq^2 -p^2+p^2q-q\\&+2pq-p^2q) + \alpha ^2\epsilon _- ^2(q^2p-2qp+p-pq^2+q^2-q+2pq-p^2q-p^2+p^2q)\\&\;\;\;\;+2[q-p][-pq\alpha ^2(1-\epsilon _+ ) \epsilon _- +{\bar{p}}{\bar{q}}\gamma ^2(1-\epsilon _+ )\epsilon _- \\&\;\;\;\;\;\;\;\;\;\;\;\;\;\;\;\;\;\;\;-pq\alpha \gamma (1-\epsilon _+ )^2+ {\bar{p}}{\bar{q}}\alpha \gamma \epsilon _- ^2]]\\ =&\rho ^2[(1-\epsilon _+ )^4pq(q-p)-\epsilon _- ^4{\bar{p}}{\bar{q}}(q-p)+(1-\epsilon _+ )^2\epsilon _- ^2(1-p-q)(p-q)\\&\;\;+2qp(1-\epsilon _+ )^3\epsilon _- (p-q)+2{\bar{p}}{\bar{q}}(1-\epsilon _+ )\epsilon _- ^3(q-p)]\\&+{\bar{\rho }}^2[\alpha ^4pq(q-p)+\gamma ^4{\bar{p}}{\bar{q}}(p-q)+\alpha ^2\gamma ^2(1-p-q)(p-q)\\&\;\;\;\;\;+2qp\alpha ^3\gamma (p-q)+2{\bar{p}}{\bar{q}}\alpha \gamma ^3(q-p)]\\&+\rho {\bar{\rho }}[2(q-p)\epsilon _- ^2\gamma ^2[s^2r^2pq -{\bar{p}}{\bar{q}}]\\&\;\;\;\;+[\gamma ^2(1-\epsilon _+ )^2+\alpha ^2\epsilon _- ^2][q^2-p^2+p-q]\\&\;\;\;\;+2[q-p]\gamma ^2\epsilon _- ^2[-pqs^2r+{\bar{p}}{\bar{q}}r-pqsr^2+ s{\bar{p}}{\bar{q}}]]\\ =&{\rho ^2(q-p)}{\epsilon _- ^4}[r^4pq-{\bar{p}}{\bar{q}}+r^2\epsilon _- ^2(p+q-1)-2qpr^3+2{\bar{p}}{\bar{q}}r]\\&+{{\bar{\rho }}^2(q-p)}{\gamma ^4}[s^4pq-{\bar{p}}{\bar{q}}+r^2\gamma ^2(p+q-1)-2qpr^3+2{\bar{p}}{\bar{q}}r]\\&+\rho {\bar{\rho }}[(q-p)\epsilon _- ^2\gamma ^2(2s^2r^2pq -2{\bar{p}}{\bar{q}}+[r^2+s^2][q+p-1])\\&\;\;\;\;+2[q-p]\gamma ^2\epsilon _- ^2(r+s)[{\bar{p}}{\bar{q}}-pqrs]]\\ =&{\rho ^2(q-p)}{\epsilon _- ^4}[r^2pq-{\bar{p}}{\bar{q}}][r-1]^2+{{\bar{\rho }}^2(q-p)}{\gamma ^4}[s^2pq-{\bar{p}}{\bar{q}}][s-1]^2\\&+\rho {\bar{\rho }}(q-p)\epsilon _- ^2\gamma ^2[2(r^2s^2pq-{\bar{p}}{\bar{q}})+[r^2+s^2][pq-{\bar{p}}{\bar{q}}] -2[r+s](rspq-{\bar{p}}{\bar{q}})]\\ =&[q-p]\cdot (\rho ^2\cdot [(1-\epsilon _+ )^2pq-\epsilon _- ^2{\bar{p}}{\bar{q}}][1-\epsilon _+ -\epsilon _- ]^2+{\bar{\rho }}^2\cdot [\alpha ^2 pq-\gamma ^2{\bar{p}}{\bar{q}}][\alpha -\gamma ]^2\\&+\rho {\bar{\rho }}\cdot [2((1-\epsilon _+ )^2\alpha ^2 pq-\epsilon _- ^2\gamma ^2 {\bar{p}}{\bar{q}})+[(1-\epsilon _+ )^2\gamma ^2+\epsilon _- ^2\alpha ^2][pq-{\bar{p}}{\bar{q}}]\\&\quad \quad \quad -2(\alpha \epsilon _- +(1-\epsilon _+ )\gamma )((1-\epsilon _+ )\alpha pq-\epsilon _- \gamma {\bar{p}}{\bar{q}})])\\ =&[q-p]\cdot (\rho ^2\cdot [(1-\epsilon _+ )^2pq-\epsilon _- ^2{\bar{p}}{\bar{q}}][1-\epsilon _+ -\epsilon _- ]^2+{\bar{\rho }}^2\cdot [\alpha ^2 pq-\gamma ^2{\bar{p}}{\bar{q}}][\alpha -\gamma ]^2\\&+\rho {\bar{\rho }}\cdot [ p q\cdot ((1-\epsilon _+ )^2\gamma ^2+\epsilon _- ^2\alpha ^2+2[-\epsilon _- \alpha -(1-\epsilon _+ )\gamma +(1-\epsilon _+ )\alpha ](1-\epsilon _+ )\alpha )\\&\quad \quad \quad -{\bar{p}}{\bar{q}}\cdot ((1-\epsilon _+ )^2\gamma ^2+\epsilon _- ^2\alpha ^2+2[-\epsilon _- \alpha -(1-\epsilon _+ )\gamma +\epsilon _- \gamma ]\epsilon _- \gamma )])\\ =&[q-p]\cdot \Bigl (\rho ^2\cdot ((1-\epsilon _+ )-\epsilon _- )^2\cdot \Bigl (\frac{(1-\epsilon _+ )^2}{\epsilon _- ^2}-\frac{{\bar{p}}{\bar{q}}}{pq}\Bigr )+{\bar{\rho }}^2\\&\quad \cdot (\alpha -\gamma )^2\Bigl (\frac{\alpha ^2}{\gamma ^2} -\frac{{\bar{p}}{\bar{q}}}{pq}\Bigr )\\&+\rho {\bar{\rho }}\cdot \Bigl (\frac{(1-\epsilon _+ )^2\gamma ^2+\epsilon _- ^2\alpha ^2+2[-\epsilon _- \alpha -(1-\epsilon _+ )\gamma +(1-\epsilon _+ )\alpha ](1-\epsilon _+ )\alpha }{(1-\epsilon _+ )^2\gamma ^2+\epsilon _- ^2\alpha ^2+2[-\epsilon _- \alpha -(1-\epsilon _+ )\gamma +\epsilon _- \gamma ]\epsilon _- \gamma }\\&\quad -\frac{{\bar{p}}{\bar{q}}}{p q}\Bigr )\Bigr ) . \end{aligned}$$

$$\square $$

## Footnotes

1 We shall here not touch on the related question on how computer simulations differ from experimentation Parker, forthcoming.

2 Several kinds of RA are distinguished in the literature. For instance, Woodward (2006) distinguishes *derivational* robustness, viz. insensitivity of the results of the inference across alternative background assumptions, from *inferential* robustness, viz. the insensitivity of the results of the inference to alternative specifications, and from *measurement* robustness, viz. agreement of measurement results across measurement techniques. We will only be concerned with derivational RA, henceforth “RA”.

3 See Currie (2018) on the epistemic advantages of models and simulations in paleontology and archaeology.

4 See the contributions to (Dardashti et al., 2019) for an in-depth discussion of the various controversial methodologies used to confirm inaccessible target systems in fundamental physics.

5 There exists no exhaustive list of such facts. For partial lists, see Cont (2001) and Chakraborti et al. (2011).

6 In particular, the modellers show that the stylized facts do not depend on the behaviour of the exogenous force, that is, the changes in fundamental value, because fundamental value changes and (absolute) returns have different scaling properties (see Lux & Marchesi, 1999, p. 499).

7 The model by Arthur et al. purports to reproduce yet another fact, namely trading volume oscillations. This difference is, however, irrelevant to our argument.

8 This entails that neither model explains stylized facts by “isolating” heterogeneity.

9 See Proposition 3 and Theorem 1 for our Bayesian treatment of conflicting evidence.

10 Notice that the difference $P(h_i|x)-P(h_i|{\bar{x}})$ could be rather small, but as long as it is strictly positive—which it is here—the $H_i$ are testable consequences of *X* in the sense of (Bovens & Hartmann, 2003).

11 Our auxiliary variable corresponds to the reliability variable in Bovens & Hartmann’s (2003) model (see also Merdes et al., 2021).

12 As documented by Samanidou et al. (2007) and Chakraborti et al. (2011), recent models improve on the first, pioneering models in that they—among other things—capture more and more of these stylized facts.

13 This is demonstrated in the proof of Theorem 1.

14 The level of empirical validation can vary: one may wish to recover the qualitative behaviour of empirical macro structure, the quantitative behavior of macro structure or the quantitative behaviour of micro structure (see, e.g., Barde & van der Hoog, 2017).

15 Propositions 1 and 2 are instances of Theorem 1 ($n=1$ and $n=2$, $m=0$) which is proved in Appendix 1.

16 Stegenga and Menon’s own goal is to qualify—in a Bayesian framework—the thesis that robustness requires “various” evidence. They explicitly say (2017, fn. 2) that they are not concerned with derivational RA. We, by contrast, discuss how the same kind of evidence, as obtained by models making different background assumptions, is confirmatory, that is, we discuss derivational RA. In spite of the different motivation, Stegenga and Menon’s argument to the point that *d*-separation is necessary to robustness directly threatens our conclusion.

17 It is well known that accumulating more propositions which are individually confirmatory need not result in more confirmation when updating upon all the available evidence (Carnap, 1962, p. 382). So, the mere accumulation of (dis)confirmatory evidence does not entail increased (dis)confirmation when updating on growing bodies of evidence.

18 The degree to which the models make non-varied assumptions is the degree to which the content of $A'$ covers the content of $A'\cup A_i$.

19 Upshot 1 and Upshot 3a differ in that they relate to different problems. For Upshot 1 one considers the addition further HET models and for Upshot 3a one sticks to two HET models but gradually increases the independence of the auxiliaries.

20 Mathematically, this linear convex combination just amounts to a linear combination of the relevant inequality (cf. Landes, 2021 for details).
